# Cardiac Contractility Structure-Activity Relationship and Ligand-Receptor Interactions; the Discovery Of Unique and Novel Molecular Switches in Myosuppressin Signaling

**DOI:** 10.1371/journal.pone.0120492

**Published:** 2015-03-20

**Authors:** Megan Leander, Chloe Bass, Kathryn Marchetti, Benjamin F. Maynard, Juan Pedro Wulff, Sheila Ons, Ruthann Nichols

**Affiliations:** 1 Undergraduate Biochemistry Program, Chemistry Department, University of Michigan, Ann Arbor, Michigan, United States of America, 48109; 2 Biological Chemistry Department, University of Michigan Medical School, Ann Arbor, Michigan, United States of America, 48109; 3 Undergraduate Chemistry Program, Chemistry Department, University of Michigan, Ann Arbor, Michigan, United States of America, 48109; 4 Laboratorio de Genética y Genómica Funcional, Centro Regional de Estudios Genómicos, Facultad de ciencias Exactas, Universidad Nacional de La Plata, Bv. 120 1459, Buenos Aires, Argentina; University of Hong Kong, HONG KONG

## Abstract

Peptidergic signaling regulates cardiac contractility; thus, identifying molecular switches, ligand-receptor contacts, and antagonists aids in exploring the underlying mechanisms to influence health. Myosuppressin (MS), a decapeptide, diminishes cardiac contractility and gut motility. Myosuppressin binds to G protein-coupled receptor (GPCR) proteins. Two *Drosophila melanogaster* myosuppressin receptors (DrmMS-Rs) exist; however, no mechanism underlying MS-R activation is reported. We predicted DrmMS-Rs contained molecular switches that resembled those of Rhodopsin. Additionally, we believed DrmMS-DrmMS-R1 and DrmMS-DrmMS-R2 interactions would reflect our structure-activity relationship (SAR) data. We hypothesized agonist- and antagonist-receptor contacts would differ from one another depending on activity. Lastly, we expected our study to apply to other species; we tested this hypothesis in *Rhodnius prolixus*, the Chagas disease vector. Searching DrmMS-Rs for molecular switches led to the discovery of a unique ionic lock and a novel 3–6 lock, as well as transmission and tyrosine toggle switches. The DrmMS-DrmMS-R1 and DrmMS-DrmMS-R2 contacts suggested tissue-specific signaling existed, which was in line with our SAR data. We identified *R*. *prolixus* (Rhp)MS-R and discovered it, too, contained the unique myosuppressin ionic lock and novel 3–6 lock found in DrmMS-Rs as well as transmission and tyrosine toggle switches. Further, these motifs were present in red flour beetle, common water flea, honey bee, domestic silkworm, and termite MS-Rs. RhpMS and DrmMS decreased *R*. *prolixus* cardiac contractility dose dependently with EC_50_ values of 140 nM and 50 nM. Based on ligand-receptor contacts, we designed RhpMS analogs believed to be an active core and antagonist; testing on heart confirmed these predictions. The active core docking mimicked RhpMS, however, the antagonist did not. Together, these data were consistent with the unique ionic lock, novel 3–6 lock, transmission switch, and tyrosine toggle switch being involved in mechanisms underlying TM movement and MS-R activation, and the ability of MS agonists and antagonists to influence physiology.

## Introduction

Peptidergic signaling plays numerous critical roles in transmitting and regulating physiological processes. Therefore, delineating the mechanisms that underlie these events is a powerful approach to identifying target molecules to influence health. An important first step in signaling is when a ligand binds to a G protein-coupled receptor protein (GPCR). Ligand-receptor binding disrupts molecular switches which cause TM movement and ultimately results in receptor activation reviewed in [1].

Myosuppressin dramatically decreases cardiac contractility and gut motility [2, 3]. First isolated as a cockroach brain peptide that affects spontaneous contractions of the gut, myosuppressin was subsequently found to be distributed throughout the invertebrates. The conservation of its structure and activities, and its widespread distribution are consistent with myosuppressin playing an important role in physiology.

Myosuppressins are members of a family of peptides with an identical C-terminal RF-NH_2_, however, the N-terminal extension is unique and differs in length and sequence [4]. The identical C terminus and variant N terminus are both important in binding and activating signaling pathways [5, 6]. FMRF-NH_2_, the first RF-NH_2_-containing peptide isolated, was identified from a neural extract applied to a clam heart preparation [7]. This superfamily of peptides is grouped based on XRF-NH_2_, where myosuppressins often contain X = L. Myosuppressins are typically represented by X1DVX2HX3FLRF-NH_2_, where X1 = pQ, P, T, A; X2 = D, G, V; X3 = V, S [8].

*Drosophila melanogaster* myosuppressin (DrmMS; TDVDHVFLRF-NH_2_) is representative of its peptide family [9]. DrmMS is pleotropic decreasing the frequency of both cardiac contractility and gut motility. The DrmMS structure-activity relationship (SAR) for its effect on cardiac contractility and gut motility is reported; the data are consistent with DrmMS having distinct signaling pathways in heart and gut [8]. In addition, DrmMS binds to two putative GPCR proteins, DrmMS-R1 and DrmMS-R2 [10]. Apart from these facts, little is known about MS signaling. No mechanism which underlies MS receptor activation, a crucial step in signal transduction, is described in literature. And, the design and characterization of MS antagonists in a disease vector are molecularly and physiologically limited in scope in the literature.

The kissing bug, *Rhodnius prolixus*, is a vector of the Chagas disease, an important health problem [11]. RhpMS, pQDIDHVFMRF-NH_2_, contains two substitutions compared to the MS consensus structure; V3 → I3 and L8 → M8 [12]. The physicochemical characteristics of myosuppressins are conserved with the residue replacements [12, 13]; RhpMS affects *R*. *prolixus* heart rate [14]. The unique MS provides an opportunity to further explore a pathway that affects a crucial physiological function in a disease vector. Previously, little was known about RhpMS signaling; its receptor sequence and structure was unidentified and its SAR uncharacterized.

This study tested our prediction that MS signaling would mimic mechanisms involved in Rhodopsin activation. We searched for molecular switch motifs across DrmMS-Rs and discovered the unique ionic lock and novel 3–6 lock, in addition to the transmission and tyrosine toggle switches. We also tested our belief that DrmMS-DrmMS-R1 and DrmMS-DmrMS-R2 interactions would reflect our SAR data, which it did. When DrmMS and its N-terminal truncation and alanyl-substituted analogs were docked to the DrmMS-Rs, the ligand contacts were distinct between receptors consistent with our SAR data. DrmMS interactions with DrmMS-R2 mirrored the cardiac contractility SAR data and DrmMS-DrmMS-R1 interactions reflected the gut motility data. Additionally, we hypothesized agonist- and antagonist-receptor contacts would differ from one another reflecting activity and inactivity. The docking data confirmed this prediction; agonists mirrored DrmMS interactions, yet an inactive analog failed to mimic parent peptide contacts.

Lastly, we expected our study to apply to other species; we tested this hypothesis in *R*. *prolixus*. We investigated RhpMS-R structure, binding pockets, ligand contacts, and SAR. We identified the *R*. *prolixus* receptor and found it shared substantial sequence identity to DrmMS-R1 and DrmMS-R2, 56% and 51%, respectively. The predicted protein was modeled to find the receptor contained typical GPCR features. RhpMS-R contained the unique myosuppressin ionic lock and novel 3–6 lock, and the transmission and tyrosine toggle switches, which, upon ligand binding, promoted TM movement and receptor activation. We obtained further support for the role of the unique and novel locks by identifying and modeling the red flour beetle, common water flea, honey bee, domestic hornworm, and termite MS-Rs; their structures, too, contained the myosuppressin motifs, which were likely involved in TM movement and receptor activation.

Due to the conservation of physicochemistry of the amino acids, which differed between the peptides and the high receptor sequence identity, we predicted RhpMS and DrmMS would be alike in activity and ligand contacts. RhpMS and DrmMS decreased *R*. *prolixus* cardiac contractility dose dependently with EC_50_ values of 140 nM and 50 nM, respectively. Based on ligand-receptor contacts, analogs were predicted to be an RhpMS agonist or inactive and act as an antagonist. [5–10]RhpMS mimicked the full-length peptide; [6–10]RhpMS applied to *R*. *prolixus* heart was inactive and blocked the effect of the parent peptide. Together, data from these studies confirmed tissue specificity in MS signaling, and supported the roles of a unique and a novel lock in MS-R activation.

## Materials and Methods

### Ethics statement

This research utilized an invertebrate, *R*. *prolixus*, reared according to a protocol in complete agreement with the recommendation established the by Directive 2010/63/EU pf the European Parliament and the Resolution 1047/2005 Annex II of the National Council of Scientific and Technical Researches (COINCET; Buenos Aires, Argentina) related to the protection of animals used for scientific purposes.

### Animals

*R*. *prolixus* were maintained at 28°C under high humidity in a cycle of 12h:12h (L:D). The insects used were 5^th^ instar 2–3 weeks post-ecdysis fed on chicken blood.

### Chemicals

All peptides were synthesized on a 433A Applied Biosystems peptide synthesizer using standard Fmoc procedures and purified by reversed-phase high performance liquid chromatography. Each synthesis was obtained with ≥95% purity and identified by matrix-assisted laser desorption/ionization time-of-flight mass spectrometry. Peptides were diluted in series with physiological saline to obtain the working solutions.

### Bioassays

Fifth instar *R*. *prolixus* were placed ventral surface down on a dish with paraffin wax and dissected under physiological saline. Segments four to seven from the dorsal abdominal cuticle were removed using minuten pins leaving the dorsal vessel exposed. The semi-intact preparation was equilibrated in 20 μl saline for 20 minutes at room temperature (25° ± 2°C), replacing the fluid with fresh saline every five minutes. Heart rate was measured under saline after the final bath, counting the number of contractions in one minute. This counting was replicated three times for each preparation, and the results of the three countings were averaged. After this, the saline was removed and replaced with an equal volume of solution containing a peptide. The heart rate was measured and expressed as % of the control (saline). Ten animals were analyzed for control and for each experiment. The investigator who performed the bioassay was unaware of the peptide structures being tested.

### Data analysis

Dose response curves were generated and the effective concentrations at half-maximal response (EC_50_) values were calculated using a non-linear regression with GraphPad Prism version 6.03. Activities of RhpMS, DrmMS, and analogs were compared to saline, set at 100%, using Single Factor ANOVA to calculate p values; significance was set at p ≤ 0.05.

### Receptor identification and modeling

RhpMS-R was identified in the *R*. *prolixus* genome database (www.vectorbase.org) using DrmMS-Rs as queries. The NIH database was searched with DrmMS-R1 and DrmMS-R2 to find the primary sequences of MS-Rs in termite, *Zootermopis nevadensis*, (Accession # KDR23127.1), the red flour beetle, *Tribolium castaneum*, (Accession # EFA01374.1), common water flea, *Daphnia pulex*, (Accession # EFx86411.1), domestic silkworm, *Bombyx mori*, (Accession # NP_001036929.1), and honey bee, *Apis mellifera* (Accession #s ACI90287.1; ACI90288.1). Alignment with DrmMS-Rs tested the likelihood a sequence represented a MS-R. The latter was also confirmed by phylogenetic analysis (Ons et al. manuscript submitted).

Receptor structures were modeled by I-TASSER using multiple threading alignments zhanglab.ccmb.med.umich.edu/I-TASSER/ [15] as described [5, 6]. The top model was submitted to ModRefiner zhanglab.ccmb.med.umich.edu/ModRefiner/ [16] after which the receptor structures were viewed in PyMOL [The PyMOL Molecular Graphics System version 1.7.0.3, Schrödinger, LLC]. The binding pocket location was considered during ligand docking boundary selection. Receptor model files were modified to include polar hydrogens and converted to the file type used for the docking procedure with AutoDockTools v 4.2 [17].

### Ligand docking and analysis

Ligand models were built in PyMOL and prepared for docking in AutoDockTools as previously described [5]. PyMOL and the molecular docking software AutoDock Vina [18] were used to dock ligands [5]. A similar method was employed to investigate peptide docking [19]. Physicochemical properties, type and proximity of ligand-receptor contacts, and pose overlap were used to evaluate the one group most likely to represent docking. Poses with strong contacts formed multiple favorable ligand-receptor interactions within reference distances [5, 20]. All poses were independently analyzed by two researchers.

## Results

### DrmMS-Rs: molecular switches, binding pockets, and ligand contacts

**Fig 1:**
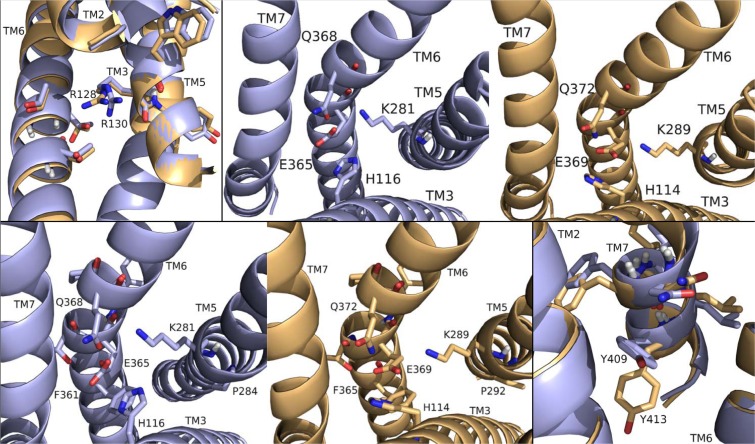
DrmMS-Rs molecular switches. DrmMS-Rs contained a unique ionic lock and novel 3–6 lock that stabilized the inactive state of the receptor. Residues involved in the ionic lock (top left), 3–6 lock (top right), transmission switch (bottom left), and tyrosine toggle switch (bottom right) are shown for DrmMS-R1 (blue) and DrmMS-R2 (gold). The receptors are shown as ribbons with TM1–7 labeled.

To begin our studies, we predicted DrmMS-Rs contained molecular switches that resembled those of Rhodopsin. DrmMS-Rs are classified as members of the Rhodopsin family A receptors [6]; as such, we expected them to contain an ionic lock, 3–7 lock, transmission switch, and tyrosine toggle switch [1]. No MS-R molecular switch was previously reported in the literature. The DrmMS-R structures contained structural motifs reminiscent of those present in Rhodopsin. The ionic lock switch was represented by the WRY motif on TM3 [Fig. 1]. It was present in the DrmMS-Rs in the same location as ERY in Rhodopsin (PDB ID: 1F88) [20]. The WRY motif of DrmMS-R interacted with multiple T residues on TM6. Even so, DrmMS-R lost an electrostatic interaction between TM3 and TM6 due to the absence of a negatively-charged residue on TM6 within the range of a salt bridge. Also, because WRY did not contain E it could not form an intra-motif interaction with R130/125 (notation used to indicate R130 in DrmMS-R1 and R125 in DrmMS-R2).

A novel 3–6 lock was also discovered. It sat between TM3 and TM6 limiting the movement of TM6 and maintaining the inactive state of the receptor. The 3–6 lock mirrored the 3–7 lock of Rhodopsin. When the lock was disrupted, TM6 likely moved and the receptor was activated [21]. TM7 in DrmMS-Rs did not contain a P residue, thus, it was more rigid than Rhodopsin and less likely to disrupt the resting state of the receptor. This was one reason stabilization of transmembrane 7 was unnecessary. TM6 was stabilized by the 3–6 lock with a salt bridge interaction between H116/114 on TM3 and E265/369 on TM6.

The DrmMS-Rs contained the TEFP transmission switch on TM6 in the same location as the CWLP motif in Rhodopsin [22]. Further, F361/365 located one helix turn away from E365/369 on TM6, H116/114 on TM3, and P284/292 located nearby on TM5 in DrmMS-Rs, were in the same position as in Rhodopsin. A notable difference was E365/369 replaced W265, which was stabilized through ionic interactions with H116/114 on TM3, K281/289 on TM5, and Q368/372 on TM7 in the DrmMS-Rs. Lastly, in the DrmMS-Rs, NFILY was similar to the Rhodopsin tyrosine toggle switch motif, NPVIY [23]. The loss of P on DrmMS-R TM7 suggested the helix would be less likely to move. TM7 movement in Rhodopsin positions Y306 to extend a hydrogen bond network from the binding pocket to the intracellular side of the receptor. A loss of DrmMS-R TM7 movement suggested Y in the toggle switch may be distanced from the hydrogen bond network. Further, in the DrmMS-Rs, S292/300 replaced Y223 on Rhodopsin TM5, which also participated in this network. Due to the distance apart, S292/300 and the ionic lock did not interact, although water-mediated bonds might exist. Either configuration would result in a less extensive hydrogen bond network than observed for Rhodopsin. Together, the presence of DrmMS-R molecular switches resembled those present in Rhodopsin, with predictably similar roles in receptor activation.

DrmMS-Rs were compared to additional Rhodopsin-like receptors to determine the uniqueness of the ionic and 3–6 locks. The inactive states of the A_2A_ adenosine (A_2A_R; PDB ID: 3EML), β_2_-adrenergic (β_2_AR; PDB ID: 3NY9), chemokine CXCR4 (CXCR4; PDB ID: 3ODU), dopamine D_3_ (D_3_R; PDB ID: 3PBL), and histamine H_1_ (H_1_R; PDB ID: 3RZE) receptors reviewed in Trzaskowski et al. were used in this comparison [24, 25, 26, 27, 28]. The ionic lock in DrmMS-Rs was similar to that in H_1_R, which formed a hydrogen bond between R125 and Q416; there was lack of evidence for an ionic lock in A_2A_R, β_2_AR, and CKCR4, and the lock in D_3_R resembled that of Rhodopsin. DrmMS-Rs were the only receptors to show the presence of a 3–6 lock, all other receptors, with the exception of A_2A_R, contained a 3–7 lock. The transmission and tyrosine toggle switches between MS-Rs and additional Rhodopsin-like receptors are highly similar.

**Fig 2:**
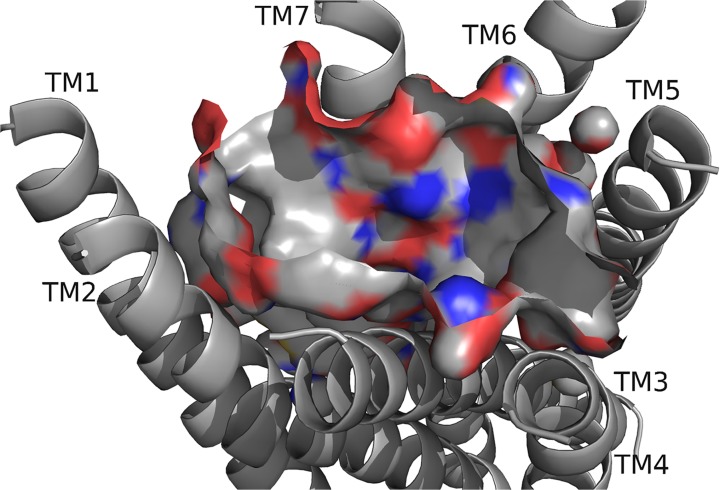
DrmMS-R1 model. The exposed atoms at the surface of the ligand-binding pocket are highlighted as carbon (gray), oxygen (red), and nitrogen (blue). The pocket contained a polar region between TM6 and TM7, and separate hydrophobic and aromatic regions near TM1 and TM5. DrmMS-R1 was undefined between TM1 and TM7, and TM4 and TM5.

**Fig 3:**
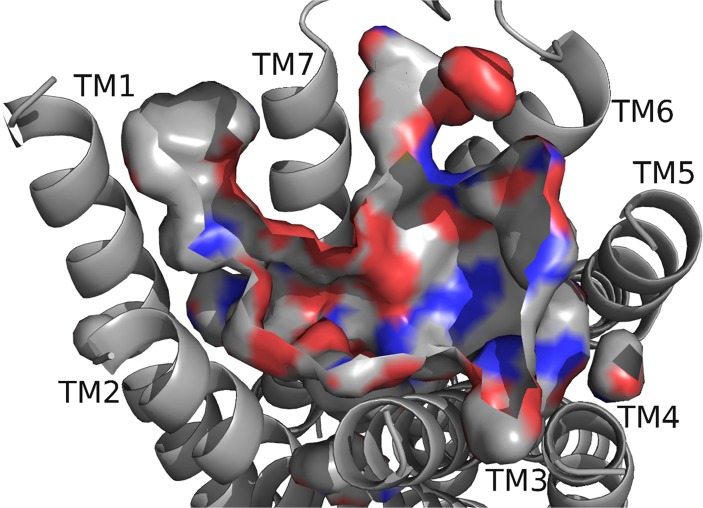
DrmMS-R2 model. The receptor backbone, binding pocket, with the exposed atoms are highlighted, and TMs are shown; refer to Fig. 2 for notation. The deepest portion of the pocket was in the region near TM5. DrmMS-R2 contained a highly polar region between TM6 and TM7 and two hydrophobic and aromatic regions near TM1 and TM5.

Next, we believed DrmMS-R1 and DrmMS-R2 interactions would reflect our SAR data [8]. This hypothesis required identifying and characterizing the DrmMS-R1 and DrmMS-R2 binding pockets. The DrmMS-R binding pockets (Fig. 2, Fig. 3) were similar to each other in shape, size, and physicochemical characteristics, but each was characterized by some unique features (Table 1, Table 2). In DrmMS-R2, W165 was between TM4 and TM5 excluding Y284 on TM5; due to its size, S167 in DrmMS-R1 did not mimic W165. Q269 in DrmMS-R1 and R277 in DrmMS-R2 differed in charge. In DrmMS-R1, K25 rotated out, yet, in DrmMS-R2, K23 was exposed resulting in a charge on TM1. In DrmMS-R1, H116 rotated toward K281 on TM6 to block access to a hydrophobic region to the center of the pocket in DrmMS-R1. Contrary, in DrmMS-R2, H114 rotated toward TM7 increasing access to this region. Y276 on TM5 in DrmMS-R1 increased the hydrophobic character, yet, the rotation of Y284 in DrmMS-R2 moved F281 further into the pocket. Together with the H114 rotation, the absence of Y284 in the pocket led to accessibility of the hydrophobic region around TM4. Also, ECL2 in DrmMS-R2 was structured with helices and beta sheets, but, in DrmMS-R1, it was predicted to have no secondary structure, making it flexible enough to be modeled between TM1 and TM7. The unique binding pockets were consistent with tissue-specific signaling as inferred by our SAR data.

**Table 1:** Residue identity differences between DrmMS-Rs.

	DrmMS-R1	DrmMS-R2
TM1	V31	F29
TM3	I110	V108
	A112	P110
TM4	S167	W165
TM5	Q269	R277
TM6	A376	V380
TM7	R389	K393

**Table 2:** Rotameric differences between DrmMS-Rs.

	DrmMS-R1	DrmMS-R2
TM1	K25	K23
	S32	S30
TM2	V73	V71
	M74	M72
	Y77	Y75
TM3	F104	F102
	I105	I103
	H108	H106
	Q113	Q111
	L115	L113
	H116	H114
	S119	S117
TM5	N270	N278
	T272	T280
	F273	F281
	Y276	Y284
	K281	K289
TM6	E365	E369
	Q368	Q372
	M371	M375
	N375	N379
TM7	S391	S395
	I396	I400
	L399	L401

**Fig 4:**
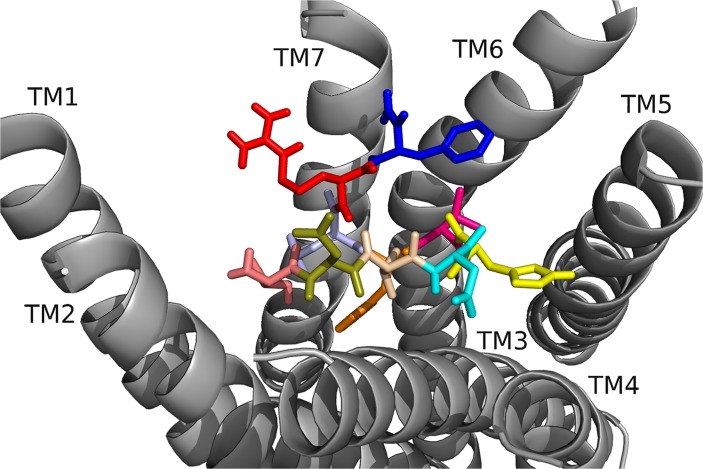
DrmMS docked to DrmMS-R1. The DrmMS ligand is shown docked to DmsMS-R1 as T1(salmon), D2 (olive), V3 (wheat), D4 (cyan), H5 (yellow), V6 (hot pink), F7 (orange), L8 (light blue), R9 (red), and F10 (blue). F7 and L8 formed a large hydrophobic network extending from the center of the pocket to TM1.

**Fig 5:**
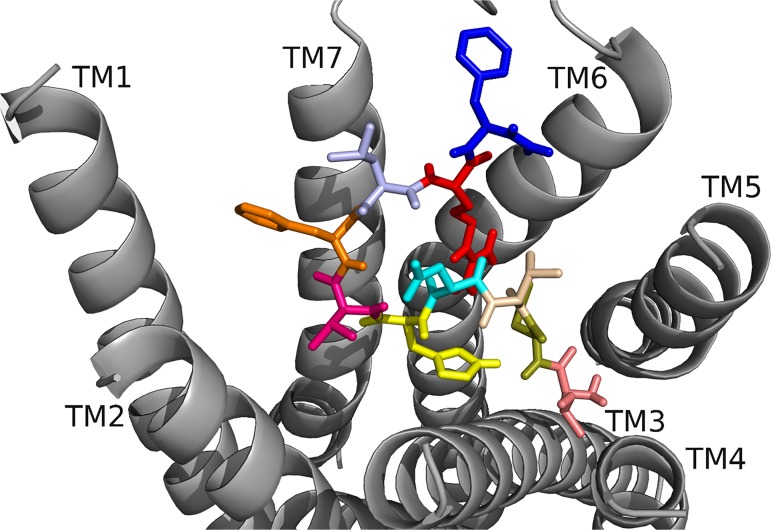
DrmMS docked to DrmMS-R2. The DrmMS ligand is shown docked to DrmMS-R2; see Fig. 4 for notation of the amino acids. Residues in the C-terminal region, particularly F7 and F10, made strong hydrophobic and aromatic contacts. D2 and R9 formed an ionic network near TM6 and the N terminus filled the remainder of the binding pocket.

[6–10]DrmMS was the heart rate active core, yet [4–10]DrmMS was the gut motility active core. The alanine scans performed in heart and gut showed the residues crucial to DrmMS activity and binding are different between the two tissues; F7 and L8 were essential in gut, and F7 and F10 in heart. We believed DrmMS-DrmMS-R1 and DrmMS-DrmMS-R2 interactions would reflect our SAR data. N-terminal truncated and alanyl-substituted analogs (Table 3) were docked to the receptors; additional data are available in Supporting Information. In DrmMS docked to DrmMS-R1 (Fig. 4, Table 4), F7 made multiple, strong hydrophobic and aromatic contacts, which L8 extended by interacting with hydrophobic residues on TM1, TM2 and TM7. H5, V6, and F10 formed additional hydrophobic contacts between TM4, TM5, and TM6; H5 made extensive interactions within this region. The N-terminal residues formed weaker hydrophobic and polar interactions to TM2 and TM3. In DrmMS-R2 (Fig. 5, Table 5), F7 formed contacts between TM1 and TM2; the position of F10 on TM7 allowed for hydrophobic and aromatic interactions with ECL3. D2 and R9 interacted and formed an ionic network near TM6. D4 made extensive intramolecular interactions with the backbone to stabilize the peptide. The N terminus spanned the pocket to make hydrophobic and polar interactions with TM3, TM4, and TM5. The residues which formed strong interactions differed between the DrmMS-Rs, in particular, L8, F7, and F10 showed receptor-specific ligand binding in line with our tissue-specific SAR data.

**Table 3:** DrmMS and analogs: structures and activities.

Peptide	Structure	Gut	Heart
DrmMS	TDVDHVFLRF-NH_2_	agonist	agonist
[A8] DrmMS	TDVDHVFARF-NH_2_	inactive	agonist
[4–10]DrmMS	DHVFLRF-NH_2_	active core	agonist
[5–10]DrmMS	HVFLRF-NH_2_	antagonist	agonist
[6–10]DrmMS	VFLRF-NH_2_	inactive	active core
[7–10]DrmMS	FLRF-NH_2_	inactive	inactive
Y-Bpa_2_DrmMS	Y-T(Bpa)VDHVFLRF-NH_2_	agonist	agonist
Y-Bpa_4_DrmMS	Y-TDV(Bpa)HVFLRF-NH_2_	agonist	antagonist

**Table 4:** DrmMS contact sites on DrmMS-R1^a^.

T	Side chain	Y77	2.9 Å
		T81	4.7 Å
		Y85	4.5 Å
		H108	1.8 Å
	Backbone	D84	2.8 Å
		D2	3.6 Å
		V3	(NH) 3.8 Å
D	Side chain	T1	3.6 Å
	Backbone	S109	4.1 Å
V	Side chain	A112	4.9 Å
		F7	4.4 Å
	Backbone	T1	(CO) 3.8 Å
D	Side chain	Q113	2.7 Å
		S167	3.8 Å
		H5	4.1 Å, (NH) 4.0 Å
	Backbone	Q113	2.2 Å
		H5	3.7 Å
H	Side chain	Q113	3.6 Å
		S167	5.1 Å
		T272	4.1 Å
		F273	5.0 Å
		Y276	2.4 Å
	Backbone	Q113	2.7 Å
		D4	4.0 Å
		F7	(NH) 3.3 Å
V	Side chain	F273	5.0 Å
		Y276	4.5 Å
		M371	4.7 Å
		F10	3.9 Å
	Backbone	Q368	2.5 Å
F	Side chain	V73	3.5 Å
		Y77	3.1 Å
		A112	2.8 Å
		H116	4.2 Å
		L399	3.9 Å
		L8	3.7 Å
	Backbone	Q368	3.8 Å
		H5	(CO) 3.3 Å
L	Side chain	Y77	3.6 Å
		I396	4.9 Å
		L399	3.8 Å
		F7	3.7 Å
	Backbone	F10	(NH) 3.3 Å
R	Side chain	Y24	3.7 Å
		D392	2.8 Å
	Backbone	—	
F	Side chain	F273	3.1 Å
		M371	2.3 Å
		G372	3.8 Å
		V6	3.9 Å
	Backbone	D392	2.1 Å
		D395	2.1 Å
		L8	(CO) 3.3 Å
NH_2_		S391	3.4 Å
		D392	2.4 Å
		D395	2.8 Å

^a^Residues numbered 1–10 are in DrmMS or RhpMS. (NH) and (CO) indicate that the residue backbone group was contacted. In the case in which a residue was contacted twice by the backbone of the same ligand residue, O and H are used to distinguish the contacts.

**Table 5:** DrmMS contact sites on DrmMS-R2^a^.

T	Side chain	Q111	3.5 Å
		T115	3.3 Å
		V161	3.3 Å
		V162	4.6 Å
		W165	4.3 Å, OH 2.7 Å
		D2	(NH) 2.9 Å, (CO) 3.7 Å
	Backbone	S285	4.1 Å
		K289	3.8 Å
D	Side chain	K289	2.8 Å
		Q372	4.1 Å
		V3	(NH) 2.4 Å
		R9	2.9 Å
	Backbone	Q111	3.6 Å
		T1	H 2.9 Å, O 3.7 Å
		H5	3.0 Å
V	Side chain	F281	3.7 Å
		G376	3.9 Å
	Backbone	H5	3.5 Å
		D2	2.4 Å
D	Side chain	H5	(NH) 3.0 Å
		V6	(NH) 2.0 Å
		F7	(NH) 3.5 Å
		L8	(NH) 4.0 Å
	Backbone	R9	2.2 Å
H	Side chain	V3	(CO) 3.2 Å
		H106	4.9 Å
		P110	3.6 Å
		D2	(CO) 3.0 Å
		H114	4.5 Å
	Backbone	Y83	3.0 Å
		D4	3.0 Å
V	Side chain	Y78	4.3 Å
		T79	4.3 Å
		H106	3.5 Å
	Backbone	Y83	3.6 Å
		D4	2.0 Å
F	Side chain	Y22	4.7 Å
		K23	4.0 Å
		H26	4.7 Å
		Y83	3.9 Å
		L8	4.4 Å
	Backbone	S395	2.7 Å
		D4	3.5 Å
L	Side chain	L392	3.6 Å
		F7	4.4 Å
	Backbone	D4	4.0 Å
R	Side chain	Q372	2.6 Å
		D399	3.7 Å
		D2	3.6 Å
		D4	(CO) 2.2 Å
	Backbone	N379	3.4 Å
		Y391	2.7 Å
		S395	3.8 Å
		NH_2_	2.4 Å
F	Side chain	F386	3.7 Å
		Y391	3.5 Å
	Backbone	—	
NH_2_		R9	(CO) 2.4 Å

^a^Residues numbered 1–10 are in DrmMS or RhpMS. (NH) and (CO) indicate that the residue backbone group was contacted. In the case in which a residue was contacted twice by the backbone of the same ligand residue, O and H are used to distinguish the contacts.

**Fig 6:**
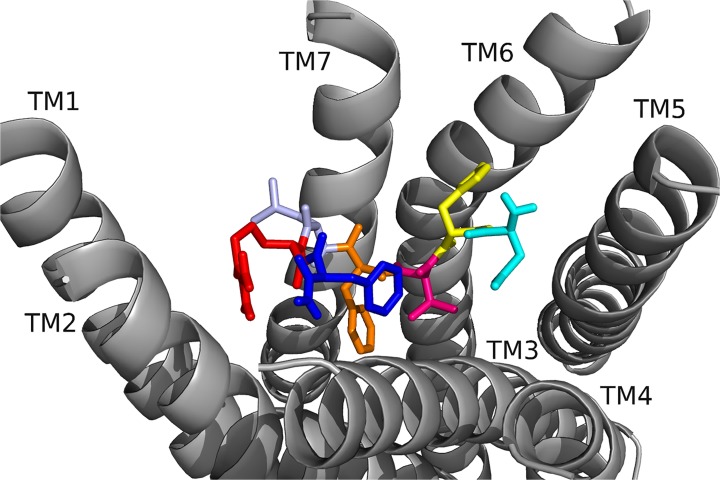
[4–10]DrmMS docked to DrmMS-R1. [4–10]DrmMS retained many of the DrmMS contact sites with DrmMS-R1 thus mimicking the parent peptide, consistent with SAR data that indicate the analog is active in heart and gut. See Fig. 4 for notation of the ligand amino acids.

**Fig 7:**
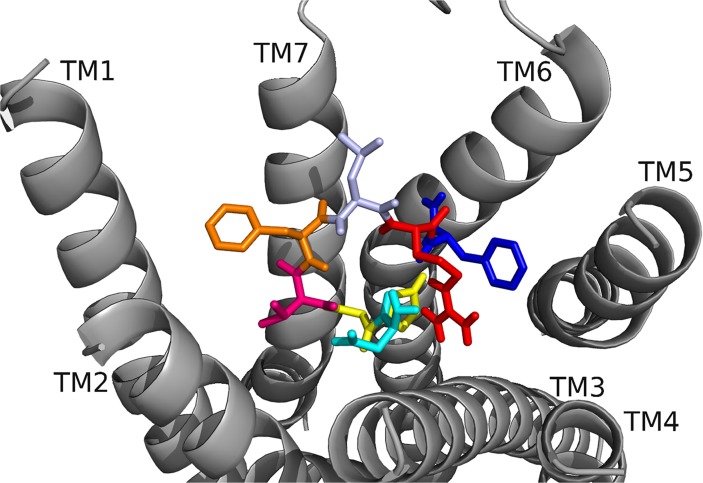
[4–10]DrmMS docked to DrmMS-R2. [4–10]DrmMS retained many of the DrmMS contact sites with DrmMS-R2, thus mimicking the parent peptide, consistent with SAR data that indicate the analog is active in heart and gut. See Fig. 4 for notation of the ligand amino acids.

In [4–10]DrmMS, active in heart and the active core in gut, docked to DrmMS-R1 (Fig. 6, Table 6), F7 initiated a hydrophobic network that was extended by L8 to the TM1, TM2, and TM7 interface. V6 and F10 contributed to this network by making contacts near TM2 and TM3, respectively. In DrmMS-R2, the analog mimicked DrmMS, though the contacts were not made by the same residues (Fig. 7, Table 7). F10 spanned the pocket to pi-stack to TM5. The C-terminal amide docked between TM6 and TM7 leading R9 to form hydrogen bonds with TM3, D4, and H5, which created a large hydrogen bond network extending to the bottom of the pocket. V6 and F7 maintained DrmMS-like contacts and interacted with each other, creating a small hydrophobic network between TM1 and TM2. The absence of three N-terminal residues did not change the backbone conformation or contacts relative to DrmMS. Thus, the contact sites between [4–10]DrmMS and the DrmMS-Rs were consistent with the analog being active in heart and gut.

**Table 6:** [4–10]DrmMS contact sites on DrmMS-R1^a^.

D	Side chain	Q113	3.2 Å
		Y276	3.6 Å
	Backbone	H5	3.3 Å
H	Side chain	F273	3.5 Å
		M371	3.6 Å
		D2	(NH) 3.3 Å
	Backbone	K281	3.5 Å
		Q368	3.1 Å
V	Side chain	A112	4.2 Å
		H116	3.7 Å
		Y276	4.9 Å
		F7	3.8 Å
		F10	3.7 Å
	Backbone	Q368	3.1 Å
F	Side chain	V73	3.5 Å
		Y77	3.7 Å
		A112	3.7 Å
		L115	4.8 Å
		H116	4.0 Å
		L399	3.7 Å
		V6	3.8 Å
	Backbone	Q368	3.7 Å
L	Side chain	H28	4.1 Å
		Y85	4.5 Å
		I396	3.6 Å
		L399	4.5 Å
	Backbone	—	
R	Side chain	D84	2.3 Å
		Y85	4.1 Å
		H108	3.0 Å
		R9	(CO) 3.2 Å
		F10	(CO) 3.0 Å
		NH_2_	3.2 Å
	Backbone	Y77	O 3.0 Å, H 3.5 Å
		H108	3.2 Å
		R9	3.2 Å
F	Side chain	Y77	4.5 Å
		H108	4.4 Å
		A112	3.8 Å
		V6	3.7 Å
	Backbone	R9	3.0 Å
NH_2_		H108	3.7 Å
		R9	3.2 Å

^a^Residues numbered 1–10 are in DrmMS or RhpMS. (NH) and (CO) indicate that the residue backbone group was contacted. In the case in which a residue was contacted twice by the backbone of the same ligand residue, O and H are used to distinguish the contacts.

**Table 7:** [4–10]DrmMS contact sites on DrmMS-R2^a^.

D	Side chain	H106	3.1 Å
		H5	(NH) 2.8 Å
		V6	(NH) 2.3 Å
	Backbone	F7	(NH) 4.1 Å
		R9	2.6 Å, (NH) 3.8 Å
		F10	(NH) 3.3 Å
H	Side chain	Y75	3.9 Å
		H106	4.0 Å
		P110	3.7 Å
		H114	3.6 Å
		Q372	3.1 Å
		R9	3.5 Å
		F10	(CO) 2.0 Å
	Backbone	Y75	2.7 Å
		H106	2.3 Å
		D4	2.8 Å
V	Side chain	T79	3.6 Å
		Y83	3.8 Å
		H106	4.9 Å
		F7	3.7 Å
	Backbone	Y83	3.3 Å
		D4	2.3 Å
F	Side chain	Y22	4.7 Å
		K23	3.9 Å
		Y83	3.6 Å
		V6	3.7 Å
	Backbone	S395	3.0 Å
		D4	(CO) 4.1 Å
L	Side chain	Y391	3.7 Å
		L392	3.6 Å
	Backbone	—	
R	Side chain	S107	3.7 Å
		Q111	2.9 Å
		D4	(CO) 2.6 Å
		H5	3.5 Å
	Backbone	N379	4.3 Å
		D399	3.9 Å
		D4	(CO) 3.8 Å
F	Side chain	W165	3.6 Å
		F281	3.6 Å
		G376	5.0 Å
	Backbone	D4	(CO) 3.3 Å
		H5	2.0 Å
NH_2_		N379	3.2 Å

^a^Residues numbered 1–10 are in DrmMS or RhpMS. (NH) and (CO) indicate that the residue backbone group was contacted. In the case in which a residue was contacted twice by the backbone of the same ligand residue, O and H are used to distinguish the contacts.

**Fig 8:**
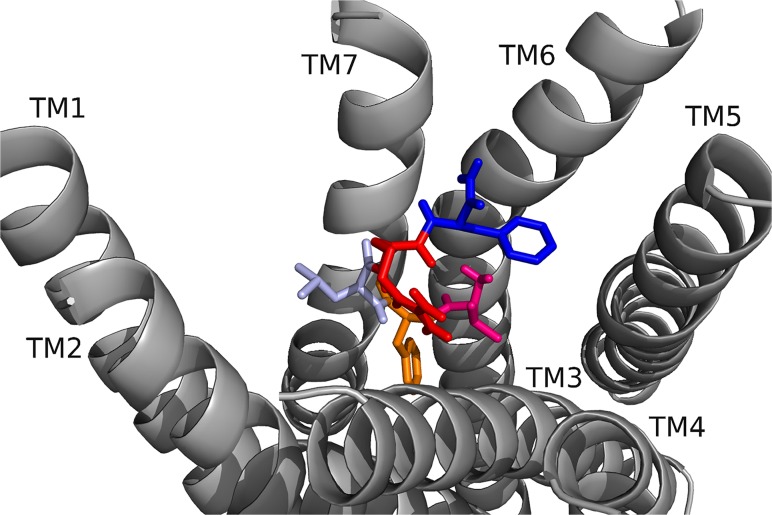
[6–10]DrmMS docked to DrmMS-R1. [6–10]RhpMS did not retain many of the DrmMS contact sites with DrmMS-R1 thus is was unlike the parent peptide, consistent with SAR data that established it was inactive in gut, but the active core in heart. Thus, taken together, these data indicated [6–10]DrmMS did activate DrmMS-R1 in the gut.

**Fig 9:**
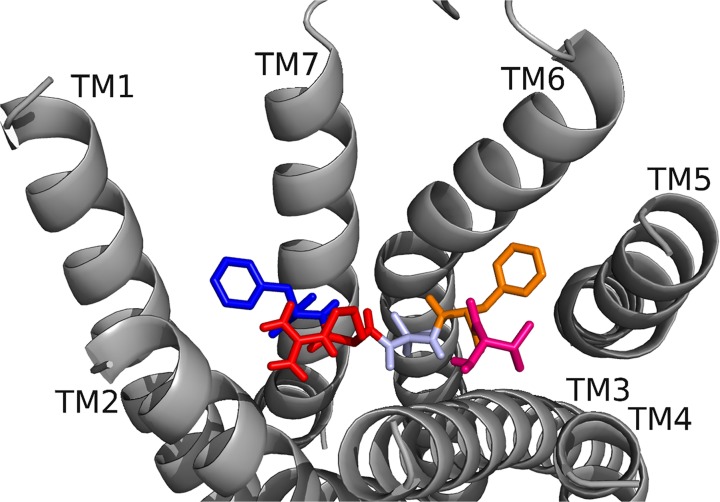
[6–10]DrmMS docked to DrmMS-R2. [6–10]DrmMS retained many of the DrmMS contact sites with DrmMS-R2, thus, mimicking the parent peptide consistent with SAR data that established it was inactive in gut, but the active core in heart. Thus, taken together, these data indicated [6–10]DrmMS may activate DrmMS-R2 in heart.

**Fig 10:**
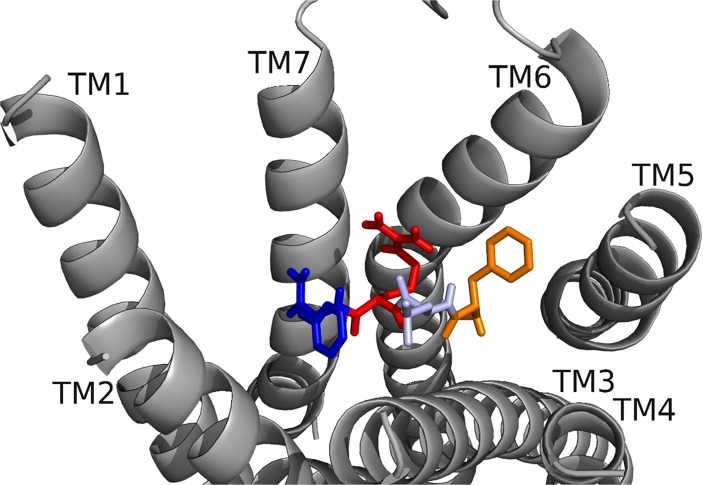
[7–10]DrmMS docked to DrmMS-R2. [7–10]DrmMS did not retain many of the DrmMS contact sites with DrmMS-R2, consistent with SAR data that indicate the analog is inactive in heart and gut. Thus, taken together, these data indicated [7–10]DrmMS, one less N-terminal residue than [6–10]DrmMS, the active core in heart, did not activate DrmMS-R2, in line with this receptor transducing the DrmMS-R2 signal.

In [6–10]DrmMS, the active core in heart, but inactive in gut, docked to DrmMS-R1 (Fig. 8, Table 8), F7 maintained multiple, strong hydrophobic and aromatic contacts, extending deeper into the pocket compared to DrmMS. L8 was directed towards TM1 and TM2 and failed to make contacts with F7. V6 made hydrophobic contacts. R9 and F10 filled the pocket yet failed to retain many DrmMS-like contacts. [6–10]DrmMS docked to DrmMS-R2 was similar to DrmMS (Fig. 9, Table 9). F10 interacted with hydrophobic and aromatic residues on TM1 and TM2. V6, F7, and L8 made hydrophobic contacts to TM3, TM4, and TM5, pi-stacked with TM5, and retained N-terminal contacts. F7 and F10 maintained strong interactions within separate networks in the pocket, much like DrmMS. The similar contacts suggested a role for DrmMS-R2 signaling in heart. In [7–10]DrmMS, inactive in heart, docked to DrmMS-R2 (Fig. 10, Table 10) the analog location was similar to [6–10]DrmMS and F7 contacts were identical. However, the loss of V6 changed the backbone conformation and altered the L8 and R9 side chain orientations and contacts. The largest difference was the rotation of F4, it contacted hydrophobic residues between TM2 and TM3, losing contacts to TM1 and TM2. [7–10]DrmMS resulted in weaker ligand-receptor contacts compared to [6–10]DrmMS, consistent with inactivity versus activity of these analogs in heart.

**Table 8:** [6–10]DrmMS contact sites on DrmMS-R1^a^.

V	Side chain	H116	3.7 Å
		T117	4.8 Å
		Y276	4.5 Å
		F10	3.5 Å
	Backbone	H116	3.3 Å
		K281	3.7 Å
		Q368	H 2.9 Å, O 3.8 Å
F	Side chain	V73	3.6 Å
		L115	4.7 Å
		H116	3.8 Å
		F361	4.7 Å
		L399	4.1 Å
	Backbone	—	
L	Side chain	Y77	3.9 Å
		T81	3.8 Å
		Y85	4.8 Å
		I396	5.0 Å
		L399	3.7 Å
	Backbone	Y77	3.6 Å
		F10	(NH) 4.0 Å
R	Side chain	Y77	3.3 Å
		Q113	3.5 Å
		R9	(CO) 2.9 Å
	Backbone	Y77	3.6 Å
		R9	2.9 Å
F	Side chain	F273	3.6 Å
		Y276	3.8 Å
		G372	5.1 Å
		V6	3.5 Å
	Backbone	Q368	4.2 Å
		D392	3.6 Å
		D395	2.4 Å
		L8	(CO) 4.0 Å
NH_2_		D392	3.9 Å
		D395	2.1 Å

^a^Residues numbered 1–10 are in DrmMS or RhpMS. (NH) and (CO) indicate that the residue backbone group was contacted. In the case in which a residue was contacted twice by the backbone of the same ligand residue, O and H are used to distinguish the contacts.

**Table 9:** [6–10]DrmMS contact sites on DrmMS-R2^a^.

V	Side chain	W165	3.8 Å
		F7	3.8 Å
	Backbone	S107	3.0 Å
F	Side chain	W165	3.5 Å
		F281	3.5 Å
		V380	5.0 Å
		V6	3.8 Å
		L8	4.3 Å
	Backbone	S107	3.6 Å
		R9	(NH) 3.7 Å
L	Side chain	Y75	5.1 Å
		H106	4.2 Å
		P110	3.9 Å
		H114	4.6 Å
		F7	4.3 Å
	Backbone	H106	2.8 Å
R	Side chain	Y78	3.7 Å
		D82	3.2 Å
	Backbone	H106	4.2 Å
		F7	(CO) 3.7 Å
F	Side chain	K23	4.4 Å
		Y83	3.7 Å
	Backbone	Y83	3.1 Å
NH_2_		Y75	3.6 Å
		Y83	3.1 Å
		D399	3.5 Å

^a^Residues numbered 1–10 are in DrmMS or RhpMS. (NH) and (CO) indicate that the residue backbone group was contacted. In the case in which a residue was contacted twice by the backbone of the same ligand residue, O and H are used to distinguish the contacts.

**Table 10:** [7–10]DrmMS ligand-receptor contact sites on DrmMS-R2^a^.

F	Side chain	W165	3.6 Å
		F281	3.6 Å
		V380	4.8 Å
	Backbone	Q111	3.7 Å
		R9	(NH) 2.5 Å
L	Side chain	I103	4.4 Å
		F10	3.7 Å
	Backbone	R9	3.1 Å
R	Side chain	Q372	3.9 Å
		D399	2.9 Å
		L8	(CO) 3.1 Å
	Backbone	Y75	3.0 Å
		H106	4.0 Å
		F7	(CO) 2.5 Å
F	Side chain	Y78	3.7 Å
		I103	3.7 Å
		H106	3.5 Å
		L8	3.7 Å
	Backbone	Y75	3.0 Å
		Y83	3.1 Å
		D399	3.9 Å
NH_2_		Y83	3.1 Å
		D399	2.9 Å

^a^Residues numbered 1–10 are in DrmMS or RhpMS. (NH) and (CO) indicate that the residue backbone group was contacted. In the case in which a residue was contacted twice by the backbone of the same ligand residue, O and H are used to distinguish the contacts.

**Fig 11:**
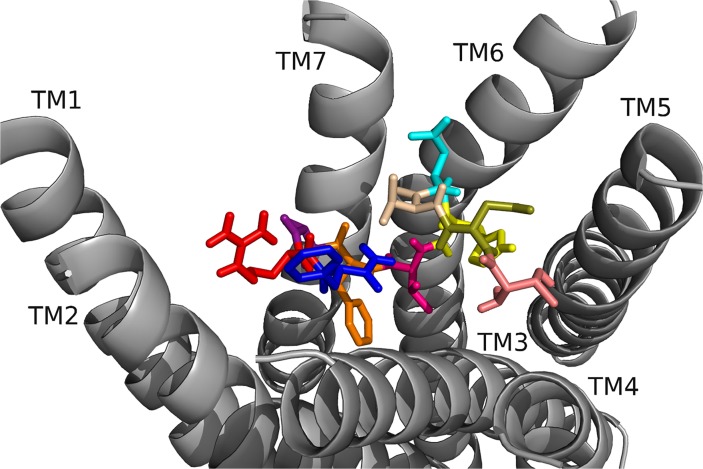
[A8]DrmMS docked to DrmMS-R1. The hydrophobic network between F7 and A8 in [A8]DrmMS was weakened in comparison to DrmMS because the branched character of the L side chain was absent. These data indicated [A8]DrmMS did not activate DrmMS-R1. A8 is purple.

**Fig 12:**
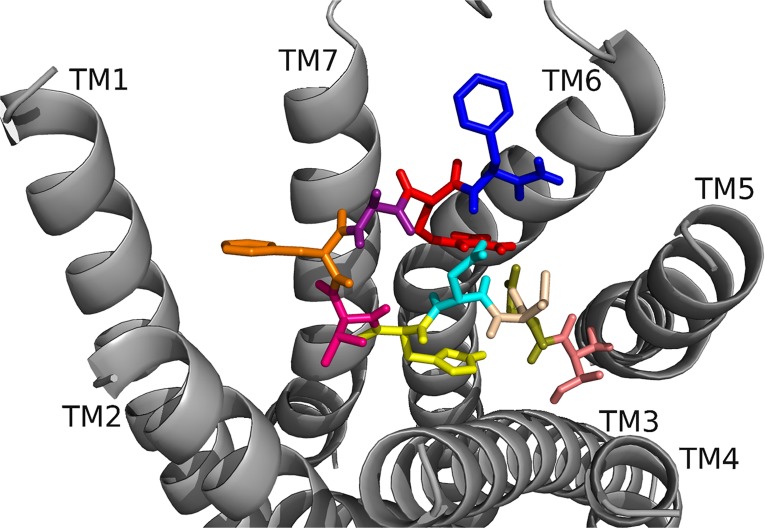
[A8]DrmMS docked to DrmMS-R2. [A8]DrmMS retained many of the DrmMS contact sites with DrmMS-R2, thus, it mimicked the parent peptide consistent with the SAR data that established it was active in heart. Thus, taken together, these data indicated [A8]DrmMS signaling involved DrmMS-R2 in heart. A8 is purple.

In [A8]DrmMS, inactive in gut, but active in heart, docked to DrmMS-R1, the C terminus was shifted relative to DrmMS (Fig. 11, Table 11). F7 formed multiple strong hydrophobic and aromatic contacts, and V6 contributed to a hydrophobic network. A8 of the analog was similarly positioned to L8, but its smaller side chain did not retain the contacts, thus, the hydrophobic network between L8, F7, the pocket bottom and the region around TM1, TM2, and TM7 were weakened. In DrmMS-R2 (Fig. 12, Table 12), [A8]DrmMS and DrmMS differed in D4 side chain orientation; D4 was rotated toward R9 increasing the ionic network in comparison to DrmMS. [A8]DrmMS contacts were consistent with the peptide signaling through DrmMS-R2 in heart.

**Table 11:** [A8]DrmMS ligand-receptor contact sites on DrmMS-R1^a^.

T	Side chain	S167	3.6 Å
		T272	3.8 Å
		H5	2.8 Å
	Backbone	Q113	2.8 Å
		S167	3.9 Å
		D2	(CO) 4.1 Å
D	Side chain	N269	3.2 Å
		D4	(NH) 3.8 Å
		H5	2.5 Å
	Backbone	H5	(NH) 3.9 Å
		NH_2_	3.5 Å
V	Side chain	F10	3.7 Å
	Backbone	V6	(NH) 4.0 Å
		NH_2_	3.8 Å
D	Side chain	Y387	2.9 Å
	Backbone	Q368	3.9 Å
		D2	3.8 Å
H	Side chain	F273	3.9 Å
		Y276	4.6 Å
		T1	2.8 Å
		D2	2.5 Å
		V6	3.6 Å, (NH) 3.7 Å
	Backbone	K281	3.2 Å
		Q368	3.4 Å
		D2	(CO) 3.9 Å
V	Side chain	H116	3.7 Å
		H5	3.7 Å
		F7	4.6 Å
	Backbone	Q368	2.9 Å
		V3	(CO) 4.0 Å
		H5	3.7 Å
F	Side chain	V73	3.6 Å
		Y77	4.1 Å
		A112	3.9 Å
		L115	4.8 Å
		H116	3.7 Å
		L399	3.5 Å
		V6	4.6 Å
	Backbone	NH_2_	3.9 Å
A	Side chain	I396	3.9 Å
		L399	4.0 Å
	Backbone	—	
R	Side chain	D84	2.6 Å
		R9	(CO) 2.3 Å
	Backbone	Y77	2.8 Å
		NH_2_	3.4 Å
F	Side chain	W101	3.7 Å
		I105	3.7 Å
		H108	3.8 Å
		S109	4.4 Å
		V3	3.7 Å
	Backbone	Y77	1.9 Å
NH_2_		D2	(CO) 3.5 Å
		V3	(CO) 3.8 Å
		R9	(CO) 3.4 Å

^a^Residues numbered 1–10 are in DrmMS or RhpMS. (NH) and (CO) indicate that the residue backbone group was contacted. In the case in which a residue was contacted twice by the backbone of the same ligand residue, O and H are used to distinguish the contacts.

**Table 12:** [A8]DrmMS ligand-receptor contact sites on DrmMS-R2^a^.

T	Side chain	T115	3.8 Å
		V161	3.6 Å
		W165	3.5 Å
		Y284	4.5 Å
		I288	4.8 Å
		D2	(NH) 2.3 Å
	Backbone	S285	3.4 Å
D	Side chain	K289	2.8 Å
		R9	2.1 Å
	Backbone	Q111	3.0 Å
		T1	2.3 Å
V	Side chain	W165	4.6 Å
		F281	3.6 Å
	Backbone	H5	3.5 Å
D	Side chain	R9	3.2 Å
		F10	(NH) 2.4 Å
	Backbone	R9	3.2 Å
H	Side chain	Y75	4.1 Å
		H106	4.1 Å
		P110	3.7 Å
		H114	3.8 Å
		V3	(CO) 3.5 Å
	Backbone	Y75	2.8 Å
V	Side chain	Y78	4.1 Å
		T79	4.8 Å
		H106	3.6 Å
	Backbone	Y83	3.8 Å
F	Side chain	Y22	4.3 Å
		K23	3.7 Å
		H26	3.9 Å
		Y83	3.7 Å
		A8	5.1 Å
	Backbone	S395	3.0 Å
A	Side chain	F7	5.1 Å
	Backbone	—	
R	Side chain	Q372	4.1 Å
		D399	4.0 Å
		D2	2.1 Å
		D4	3.2 Å, (CO) 3.9 Å
		F10	(CO) 3.9 Å
	Backbone	N379	3.2 Å
		Y391	2.8 Å
		S395	3.3 Å
F	Side chain	Y391	3.5 Å
	Backbone	N379	3.3 Å
		D4	2.4 Å
		R9	3.9 Å
NH_2_		—	

^a^Residues numbered 1–10 are in DrmMS or RhpMS. (NH) and (CO) indicate that the residue backbone group was contacted. In the case in which a residue was contacted twice by the backbone of the same ligand residue, O and H are used to distinguish the contacts.

Docking agonists and antagonists identified contacts required to bind a ligand and to activate a receptor. In DrmMS and [4–10]DrmMS docked to DrmMS-R1, H116 in the MS-R 3–6 lock may rotate toward TM7 to strengthen the network formed by F7 and L8 thus weakening the salt bridge between TM3 and TM6 and disrupting the DrmMS-R1 3–6 lock allowing receptor activation. However, in [6–10]DrmMS, F7 docked deeper in the pocket and did not allow H116 to rotate toward TM7 or disrupt the 3–6 lock, preventing TM6 movement and receptor activation. The position of F7 in [A8]DrmMS allowed the rotation of H116 toward TM7, yet the F7 and L8 network was greatly weakened, and the hydrophobic contacts extending up to TM7 were absent, consistent with the lack of receptor activation.

When docked to DrmMS-R2, DrmMS, [4–10]DrmMS, and [A8]DrmMS contacted H114, K289, and Q372, receptor residues comprising and interacting with the 3–6 lock. H114 rotated to the extracellular side of the receptor to make strong contacts with the ligand, distancing it from E369 and weakening the 3–6 lock. [6–10]DrmMS lacked polar interactions to the 3–6 lock and surrounding residues. However, L8 interacted with H114 inducing it to rotate and form stronger contacts, weakening the 3–6 lock. Further, [6–10]DrmMS retained F7 contacts of DrmMS to TM1 and TM2. Finally, [7–10]DrmMS lacked interactions with H114, K289, and Q372 and the hydrophobic residues on TM1 and TM2. Agonists and antagonist interactions with DrmMS-R2 were consistent with the SAR data in heart. Docking of agonists versus antagonist indicated that contacts to H114, K289, and Q372 and residues on TM1 and TM2 were crucial for receptor activation.

### RhpMS: receptor, molecular switches, ligand contacts, and SAR

**Fig 13:**
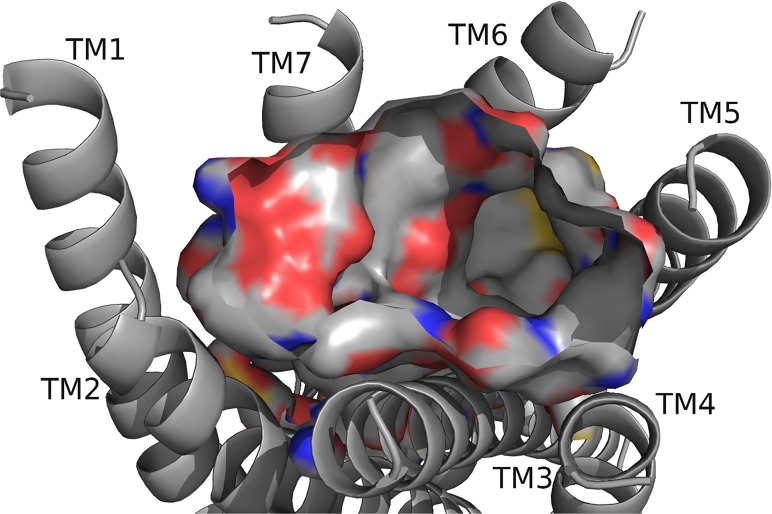
RhpMS-R model. The RhpMS-R backbone and the exposed atoms at the surface of the binding pocket are highlighted; refer to Fig. 2 for notation, including sulfur represented as yellow. The pocket contained a highly polar region near TM7 and hydrophobic and aromatic regions near TM5 and TM3. Residues on TM1 were inaccessible for ligand interactions and the pocket was deepest between TM5 and TM6.

We identified RhpMS-R in the *R*. *prolixus* genome database (www.vectorbase.org) using DrmMS-Rs as queries; it was contained in a single exon gene, Supercontig GL56309. RhpMS-R shared 56% sequence identity with DrmMS-R1 and 52% with DrmMS-R2. The hydrophobic and aromatic residues on TM2 and TM3 created a region analogous to that seen in the DrmMS-Rs (Fig. 13). K218 on TM5 was rotated out compared to DrmMS-R1 and DrmMS-R2. F210 and Y213 on TM5 were available to make strong aromatic interactions. Finally, residues on TM6 and TM7, including E281, Q284, D308, and D311, created a polar region that included a nearby residue, H114.

**Fig 14:**
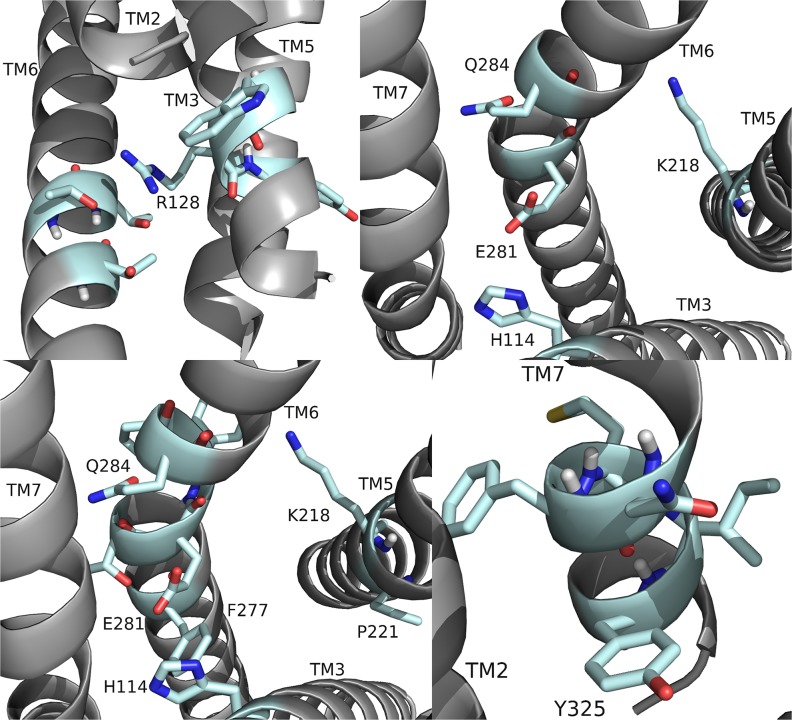
RhpMS-R molecular switches. RhpMS-R contained a unique ionic lock and novel 3–6 lock (shown) similar to DrmMS-Rs. Receptor residues involved in the ionic lock (top left), 3–6 lock (top right), transmission switch (bottom left), and tyrosine toggle switch (bottom right) are shown for RhpMS-R.

RhpMS-R was searched to identify molecular switches; it contained a unique ionic lock represented by a WRY motif on TM3 (Fig. 14). R128 of RhpMS-R formed a hydrogen-bond network with multiple T residues on TM6. The ionic lock did not form an electrostatic interaction due to the lack of a negatively charged residue on TM6 or within the WRY motif. The novel 3–6 lock was also present in RhpMS-R. Similar to DrmMS-Rs, RhpMS-R did not contain a P residue on TM7, indicating that stabilization of TM7 through a 3–7 lock would not be necessary. Rather, the 3–6 lock of RhpMS-R involved the formation of a salt bridge between H114 on TM3 and E281 on TM6, limiting the movement of TM6 and stabilizing the inactive state of the receptor. Receptor activation would involve the disruption of the 3–6 lock to allow for the movement of TM6.

**Fig 15:**
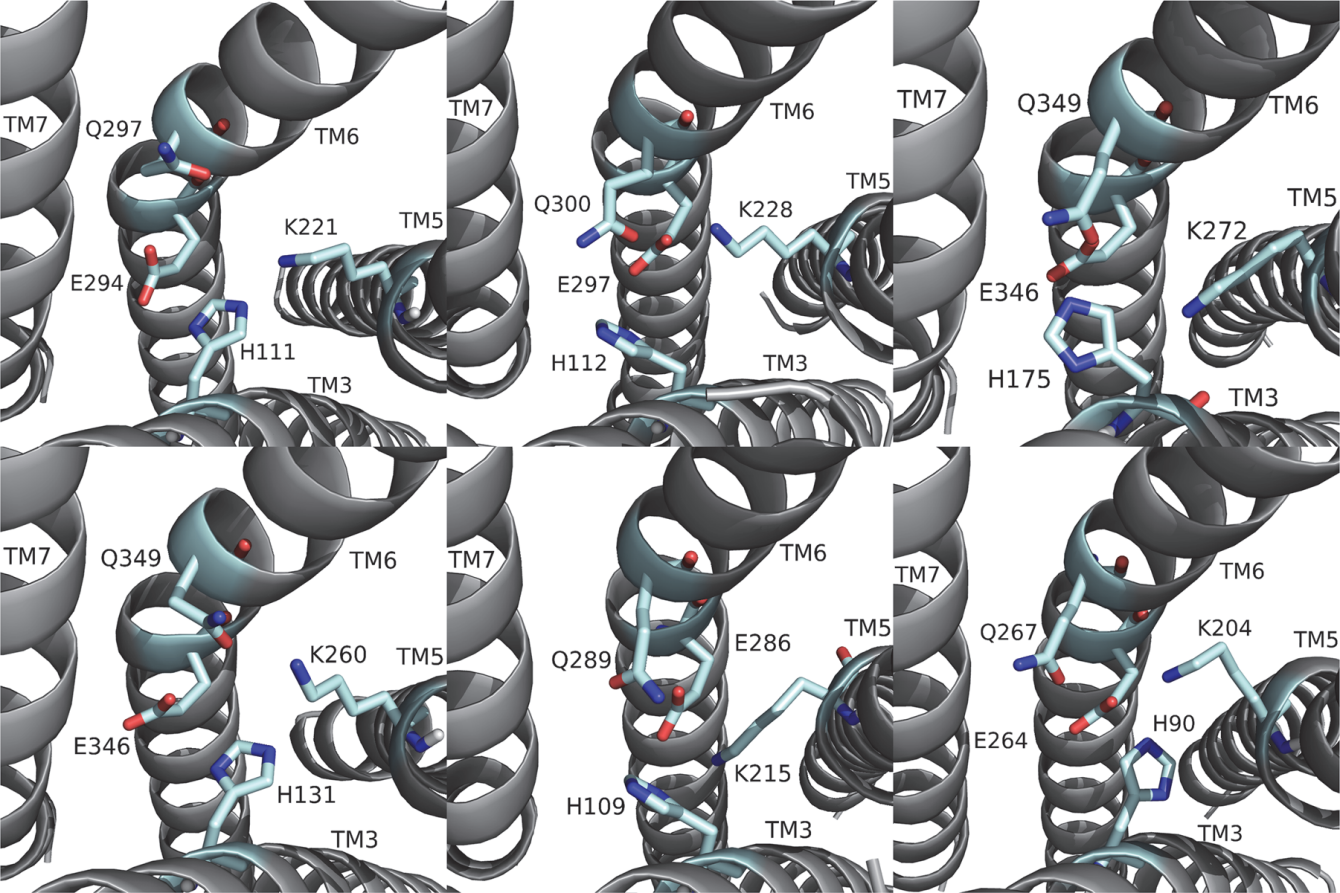
The 3–6 locks of domestic silkworm, honey bee, common water flea, red flour beetle, and termite MS-Rs. The 3–6 lock in (top: left to right) BomMS-R, ApmMS-R1, ApmMS-R2, (bottom) DapMS-R, TrcMS-R, and ZonMS-R. The receptor residues on TM5 and TM6 that interacted with the 3–6 lock are shown.

RhpMS-R contained a TEFP motif on TM6 identical to that of the DrmMS-Rs (Fig. 14). The polar character of the RhpMS-R pocket favored the presence of E281 in comparison to W265 in Rhodopsin. Further, E281 was stabilized through the 3–6 lock with an ionic interaction to H114. The conservation of the motif and presence of F227 on TM6, H114 on TM3, and P221 on TM5 indicated that the function of the transmission switch, inducing receptor activation through TM3-TM5-TM6 structural changes upon agonist binding [22], was maintained. The NFMIY motif on TM7 in RhpMS-R was structurally similar to NFILY in DrmMS-Rs, its tyrosine toggle switch. The lack of a P residue in TM7 suggested that Y325 was moved from a hydrogen bond network to the intracellular side of the receptor upon activation. Further, RhpMS-R contained S229 on TM5 which was unable to reach the hydrogen bond network. Additional work to model and search *B*. *mori* (BomMS-R), *A*. *mellifera* (ApmMS-R1 and ApmMS-R2), *D*. *pulex* (DapMS-R), *T*. *castaneum* (TrcMS-R), and *Z*. *nevadensis* (ZonMS-R) showed a conservation of the 3–6 lock within the MS-Rs (Fig. 15).

**Fig 16:**
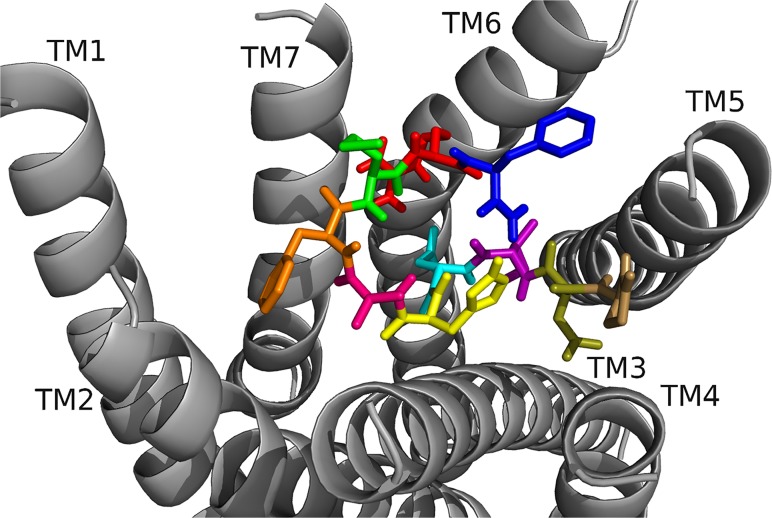
RhpMS docked to RhpMS-R. The ligand residues are pQ1(sand), D2 (olive), I3 (purple), D4 (cyan), H5 (yellow), V6 (hot pink), F7 (orange), M8 (green), R9 (red), and F10 (blue). F7 and F10 made strong hydrophobic contacts, D4 and R9 formed an ionic network near TM6, and the N terminus made general interactions to TM3, TM4, and TM5.

In RhpMS docked to RhpMS-R (Fig. 16, Table 13) F7 and F10 interacted with hydrophobic and aromatic regions, and R9 formed a salt bridge between TM6 and TM7. D4 made strong contacts, forming salt bridge interactions and extending an ionic network initiated by R9. H5 interacted extensively with the backbone and C-terminal amide to stabilize the ligand. I3, H5, V6, M8 and R9 contacts in RhpMS resembled those of DrmMS docked to DrmMS-R2. However, RhpMS was slightly shifted; Y391 on TM7 that pi-stacked with DrmMS F10 was modeled as part of ECL3 in RhpMS-R, which caused F10 to pi-stack with F210 on TM5. K23 on TM1 of DrmMS-R2 was R25 in RhpMS-R, which altered the orientations of nearby residues and resulted in F7 docking between TM2 and TM3 instead of TM1 and TM7 as in DrmMS. K218 on TM5, analogous to K289 on DrmMS-R2 that contacted D2 in DrmMS, was outside of the pocket in RhpMS-R and, thus, was unable to dock favorably. However, D4 was rotated and interacted with R9 maintaining an ionic network to TM7 as in DrmMS. Due to its size, pQ1 could not dock as deeply between TM3, TM4, and TM5, as T1 did in DrmMS, thus, it docked upwards between TM4 and TM5.

**Table 13:** RhpMS contact sites on RhpMS-R^a^.

pQ	Side chain	P164	4.9 Å
		L165	3.9 Å
	Backbone	N209	3.3 Å
D	Side chain	—	4.3 Å
	Backbone	H5	3.1 Å
		NH_2_	2.6 Å
I	Side chain	H114	5.0 Å
		F210	4.4 Å
		Y213	3.6 Å
	Backbone	Q111	2.4 Å
		H5	3.5 Å
		H5	(NH) 2.7 Å
		NH_2_	2.8 Å
D	Side chain	H114	3.1 Å
		R9	3.1 Å
	Backbone	Q111	3.0 Å
		H114	O 3.2 Å, H 3.8 Å
H	Side chain	Q111	3.7 Å
		D2	(NH) 3.1 Å
		I3	(NH) 3.5 Å
		V6	3.9 Å
		F10	(CO) 2.7 Å
	Backbone	T110	3.3 Å
		I3	(CO) 2.7 Å
V	Side chain	V103	4.0 Å
		H106	3.7 Å
		H5	3.9 Å
		F7	4.0 Å
	Backbone	Y77	3.1 Å
F	Side chain	Y77	4.7 Å
		V81	3.5 Å
		M84	4.1 Å
		Y85	4.3 Å
		H106	3. Å
		V6	4.0 Å
		M84	5.1 Å
	Backbone	—	
M	Side chain	G307	5.0 Å
		F7	5.1 Å
	Backbone	R9	3.0 Å
R	Side chain	D311	3.3 Å
		D4	3.1 Å
		M84	(CO) 3.0 Å
	Backbone	NH_2_	3.2 Å
F	Side chain	V206	4.4 Å
		F210	3.7 Å
		I292	3.1 Å
	Backbone	H5	2.7 Å
NH_2_		D2	(CO) 2.6 Å
		I3	(CO) 2.8 Å
		R9	(CO) 3.2 Å

^a^Residues numbered 1–10 are in DrmMS or RhpMS. (NH) and (CO) indicate that the residue backbone group was contacted. In the case in which a residue was contacted twice by the backbone of the same ligand residue, O and H are used to distinguish the contacts.

**Fig 17:**
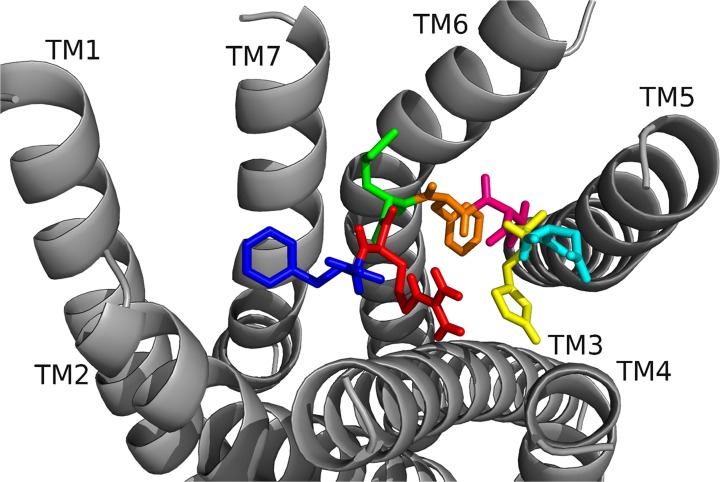
[4–10]RhpMS docked to RhpMS-R. [4–10]RhpMS differed from RhpMS in ligand orientation, yet contact sites were still retained through different ligand residues, consistent with its activity in heart.

**Fig 18:**
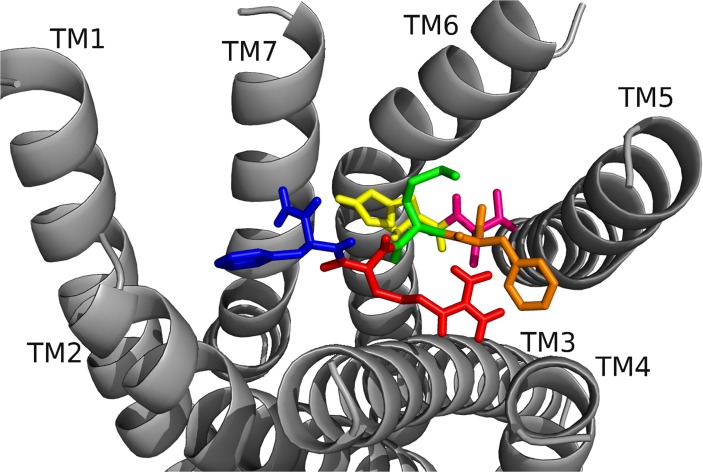
[5–10]RhpMS docked to RhpMS-R. Docking of [5–10]RhpMS was similar to that of RhpMS, consistent SAR data that indicate the analog was active.

**Fig 19:**
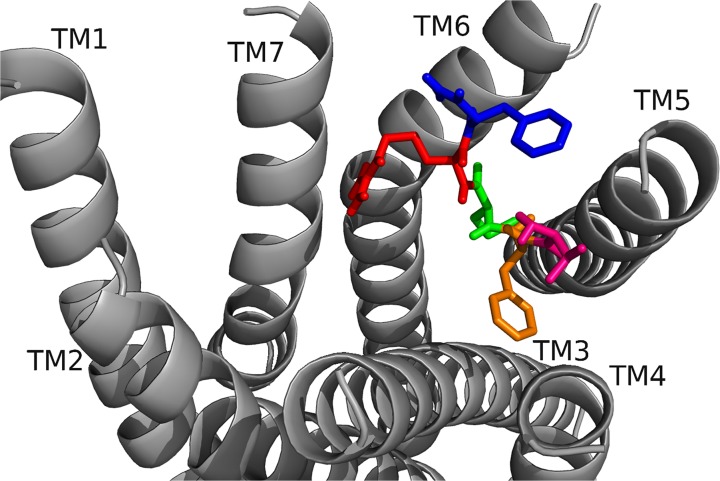
[6–10]RhpMS docked to RhpMS-R. [6–10]RhpMS retained RhpMS contacts near TM5, but lacked additional contacts of the parent peptide, consistent with its inactivity in heart.

[4–10]RhpMS (Fig. 17, Table 14) maintained most of the interactions of the parent peptide, although the contacts were not always retained by the same ligand residues. V6 and F7, and F10 of the truncated analog maintained the contacts of I3 and F7, respectively, in RhpMS. D4, H5, R9 and the C-terminal amide were involved in a large hydrogen bond network between TM3, TM4, and TM5, retaining contacts of pQ1, D2, and H5 from RhpMS. The major differences between [4–10]RhpMS and RhpMS were the lack of an ionic network between TM6 and TM7 and contacts to the top of TM5 and TM6 in [4–10]RhpMS, interactions made by D2, R9, and F10 in RhpMS. [5–10]RhpMS (Fig. 18, Table 15) also maintained many contacts of the parent peptide with its N terminus similar to [4–10]RhpMS. [5–10]RhpMS H5 and the C-terminal amide, and F10 retained the contacts of R9 and F7 of RhpMS. Although [5–10]RhpMS R9 formed hydrogen bonds to TM3 and interacted extensively with the hydrophobic region near TM5, contacts of pQ1, D2, and F10 from RhpMS were not retained. [6–10]RhpMS (Fig. 19, Table 16) docked largely to the hydrophobic region between TM4, TM5, and TM6 with little or no contacts to TM1, TM2, TM3, or TM7. A similar hydrophobic network involving V6, F7, and M8 was not as extensive as in [5–10]RhpMS. R4 extended into the binding pocket and formed a salt bridge interaction to TM7.

**Table 14:** [4–10] RhpMS contact sites on RhpMS-R^a^.

D	Side chain	N209	3.3 Å
		D4	(NH) 2.2 Å
	Backbone	D2	2.2 Å
		H5	3.2 Å
H	Side chain	Q111	3.7 Å
		C162	5.0 Å
		L165	4.4 Å
		Y213	4.4 Å
		D4	(NH) 3.2 Å
		R9	3.3 Å
	Backbone	—	
V	Side chain	F210	4.6 Å
		Y213	3.7 Å
		I217	4.4 Å
		G285	5.0 Å
		F7	3.8 Å
	Backbone	Q111	4.4 Å
F	Side chain	I118	4.1 Å
		I217	4.9 Å
		K218	4.1 Å
		G285	3.6 Å
		V6	3.8 Å
	Backbone	R9	(NH) 3.6 Å
M	Side chain	Q284	4.0 Å
		L287	4.1 Å
		G307	5.0 Å
		M310	3.7 Å
		A314	4.2 Å
		R9	(NH) 3.4 Å
	Backbone	H114	4.1 Å
		E281	3.8 Å
R	Side chain	S107	4.1 Å
		Q111	3.7 Å
		H5	3.3 Å
		F10	(CO) 2.5 Å
	Backbone	F7	(CO) 3.6 Å
		M8	3.4 Å
F	Side chain	Y77	3.6 Å
		V81	3.8 Å
		Y85	3.8 Å
		H106	4.2 Å
	Backbone	R9	2.5 Å
NH_2_		—	

^a^Residues numbered 1–10 are in DrmMS or RhpMS. (NH) and (CO) indicate that the residue backbone group was contacted. In the case in which a residue was contacted twice by the backbone of the same ligand residue, O and H are used to distinguish the contacts.

**Table 15:** [5–10] RhpMS contact sites on RhpMS-R^a^.

H	Side chain	G285	4.9 Å
		D311	3.0 Å
		A314	3.7 Å
		M8	(NH) 2.7 Å, (CO) 3.3 Å
	Backbone	H114	3.4 Å
		E281	3.7 Å
V	Side chain	F210	5.1 Å
		Y213	4.1 Å
		S214	4.4 Å
		I217	4.2 Å
		K218	3.8 Å
		G285	4.4 Å
	Backbone	Q111	2.8 Å
F	Side chain	L161	5.1 Å
		F210	4.9 Å
		Y213	3.7 Å
		R9	3.4 Å
	Backbone	R9	2.4 Å
M	Side chain	F210	3.9 Å
		F7	4.3 Å
	Backbone	Q111	4.1 Å
		H5	H 2.7 Å, O 3.3 Å
R	Side chain	F7	3.4 Å, (CO) 2.4 Å
	Backbone	Y77	3.3 Å
		NH_2_	3.6 Å
F	Side chain	Y77	4.1 Å
		V81	3.3 Å
		M84	4.5 Å
		Y85	4.0 Å
		H106	4.1 Å
	Backbone	—	
NH_2_		Y77	3.2 Å
		Y85	4.1 Å
		D311	2.6 Å
		R9	(CO) 3.6 Å

^a^Residues numbered 1–10 are in DrmMS or RhpMS. (NH) and (CO) indicate that the residue backbone group was contacted. In the case in which a residue was contacted twice by the backbone of the same ligand residue, O and H are used to distinguish the contacts.

**Table 16:** [6–10] RhpMS contact sites on RhpMS-R^a^.

V	Side chain	F7	3.9 Å
		L165	4.8 Å
		V206	4.2 Å
		F210	3.5 Å
	Backbone	—	
F	Side chain	V6	3.9 Å
		L161	5.0 Å
		L165	3.8 Å
		Y213	3.9 Å
	Backbone	—	
M	Side chain	Y213	4.3 Å
		I217	3.6 Å
		K218	3.7 Å
	Backbone	—	
R	Side chain	D311	3.1 Å
	Backbone	—	
F	Side chain	V206	3.4 Å
		F210	3.6 Å
		I292	3.7 Å
	Backbone	T291	2.4 Å
NH_2_		T291	3.0 Å

^a^Residues numbered 1–10 are in DrmMS or RhpMS. (NH) and (CO) indicate that the residue backbone group was contacted. In the case in which a residue was contacted twice by the backbone of the same ligand residue, O and H are used to distinguish the contacts.

**Fig 20:**
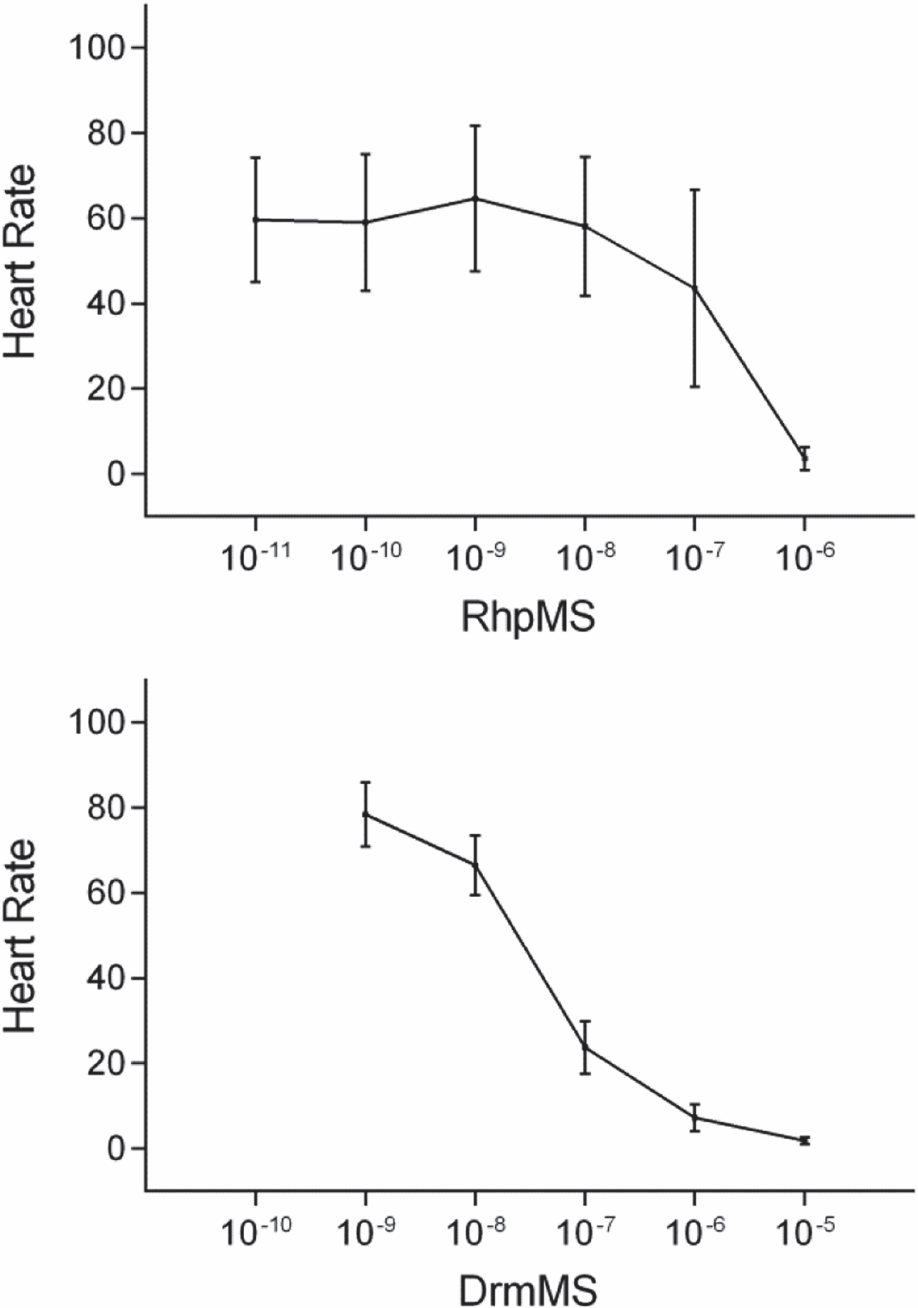
RhpMS and DrmMS dose-response curves. RhpMS dose dependently decreased *R*. *prolixus* cardiac contractility with an EC50 ∼ 140 nM. DrmMS dose dependently decreased *prolixus* cardiac contractility with an EC50 ∼ 50 nM. The y-axis is the effect of RhpMS (top) and DrmMS (bottom) on heart rate as percent of the saline response (100%). The x-axis is RhpMS or DrmMS concentration (molarity, M). Mean values ± S.E.M were reported. N = 10 animals for each experimental condition.

RhpMS and DrmMS dose dependently decreased *R*. *prolixus* heart rate with EC_50_ values of 140 nM and 50 nM, respectively (Fig. 20). The effect of N-terminally truncated analogs of RhpMS on heart rate was measured to determine the length required to elicit RhpMS activity. [4–10]RhpMS and [5–10]RhpMS docking was similar to that of RhpMS and thus these analogs were predicted to mimic the response of the parent peptide. Further, docking of [6–10]RhpMS was distinct from RhpMS and was not predicted to activate RhpMS-R. Thus, [4–10]RhpMS, [5–10]RhpMS, and [6–10]RhpMS were tested to determine their effect on heart rate.

[4–10]RhpMS decreased heart rate dose dependently, from 1 nM to 10 μM. [5–10]RhpMS and [6–10]RhpMS were measured between 10 pM and 1 μM and [6–10]RhpMS was further tested at 100 μM (S9 Fig.). At 1 μM, [4–10]RhpMS and [5–10]RhpMS were active with heart contractions 49% and 50% of saline, respectively (Table 17). [6–10]RhpMS appeared to be inactive at 1 μM with heart rate 85% of saline (Table 17). The inactive [6–10]RhpMS was competed against RhpMS to establish whether it was an antagonist. From 1 nM to 10 μM, [6–10]RhpMS was applied with 1 μM RhpMS, a concentration which elicited a potent effect, to determine if the analog antagonized the parent peptide activity. Alone, 1 μM RhpMS decreased heart rate to 4% baseline and 1 μM [6–10]RhpMS was 85%; together, the result was 38% baseline (Table 18), which indicated the analog antagonized RhpMS signaling. At lower concentrations [6–10]RhpMS was less effective, and at a 10-fold higher concentration, it was a more effective antagonist (S10 Fig.).

**Table 17:** RhpMS SAR data.

		Heart Rate	
		Mean ± S.E.M.	p value
RhpMS	pQDIDHVFMRF-NH_2_	4 ± 3	0.0
[4–10]RhpMS	DHVFMRF-NH_2_	49 ± 8	0.0
[5–10]RhpMS	HVFMRF-NH_2_	50 ± 2	0.001
[6–10]RhpMS	VFMRF-NH_2_	85 ± 11	0.7
DrmMS	TDVDHVFLRF-NH_2_	7 ± 3	0.0

The effects of RhpMS, DrmMS, and N-terminally truncated RhpMS analogs are reported as Mean ± S.E.M. at 1 μM calculated as percent of saline.

**Table 18:** RhpMS and N-terminal truncation analog competition data.

	Heart Rate	
	Mean ± S.E.M.	p value
RhpMS + [4–10]RhpMS	49 ± 10	0.004
RhpMS + [5–10]RhpMS	62 ± 9	0.02
RhpMS + [6–10]RhpMS	38 ± 18	0.001

The effects of RhpMS and an N-terminal truncated RhpMS analog applied at equal concentrations (1 μM) were reported as Mean ± S.E.M. calculated as percent of the saline response (100%).

Activation of the receptor appeared to involve disruption of the 3–6 lock between H114 and E281. D4 of RhpMS and the N-terminal amide of [5–10]RhpMS were positioned to form salt bridges with H114 and E281, respectively, weakening or disrupting the 3–6 lock and allowing TM6 to move. The ligand backbone of [4–10]RhpMS interacted minimally with H114 and E281, possibly weakening the salt-bridge interaction. Further, [4–10]RhpMS retained contact sites of RhpMS, suggesting the analog maintained interactions required for receptor activation. Conversely, [6–10]RhpMS docked to the TM5 and TM6 region, lacking interactions to the 3–6 lock and forming many interactions distinct from those of RhpMS. The lack of RhpMS contacts between TM2 and TM3 and the maintenance of the 3–6 lock are consistent with the inactivity of the analog. However, the strength of [6–10]RhpMS ligand-receptor contacts indicate favorable binding, consistent with it being an antagonist of RhpMS signaling. Docking data indicated that interactions to the 3–6 lock and hydrophobic residues between TM2 and TM3 were required for receptor activation.

## Discussion

In this paper we described molecular switches involved in TM movement that underlies MS-R activation; no prior publication reports these motifs and mechanisms. We found a unique ionic lock and novel 3–6 lock held MS-Rs in an inactive state that, upon ligand binding, may lead to TM6 movement, a crucial step for receptor activation [20, 21, 29]. A conserved TEFP present in MS-Rs mimicked the Rhodopsin transmission switch, CWLP [22, 30]. Lastly, NF(M/I)(I/L)Y in MS-Rs resembled the Rhodopsin tyrosine toggle switch motif, NPVIY.

The motifs were present and made contacts consistent with MS-R activation. Even so, the motifs of the MS-Rs were unique compared to Rhodopsin, in line with the MS peptide, receptor structures, and ligand contacts, and consistent with the DrmMS and RhpMS SAR data. The loss of contacts and networks, and weakened interactions observed in MS-Rs compared to Rhodopsin may indicate their transition from the inactive to active state occurs on a different time scale or energy level.

Myosuppressins are likely to play crucial roles in physiology; however, ligand-receptor contact data remained unpublished. DrmMS-R1 and DrmMS-R2 share high sequence identity and bind the same ligand, yet, their binding pockets differed physicochemically. The unique DrmMS-DrmMS-R1 and DrmMS-DrmMS-R2 contact data that we report and our previous SAR data [8] were consistent with tissue-specific signaling in heart and gut. Next, we explored MS ligand binding and receptor activation in *R*. *prolixus*. Although RhpMS-R shared high sequence identity with the DrmMS-Rs, its binding pocket was different in shape and size, and the residues which projected into it. These data were in line with the unique RhpMS SAR compared to DrmMS data, in particular, the differences in the RhpMS active core and antagonist structures for *R*. *prolixus* cardiac contractility.

Together, the data from this study describe molecular switches involved in receptor activation and ligand contacts which provide insight into how the motifs are involved in MS signaling. Additionally, a bioassay, and binding pockets, size and physicochemistry of the residues available to make contacts supported published data demonstrating tissue-specificity of myosuppressin signaling and yielded information on MS SAR and its receptor in a disease vector.

## Supporting Information

S1 Fig [7–10]DrmMS docked to DrmMS-R1. [7–10]DrmMS retained few contacts of DrmMS and the hydrophobic network between F7 and L8 was weakened in comparison to the parent peptide, consistent with the inactivity of the analog.
(TIF)

S2 Fig Y[Bpa2]DrmMS docked to DrmMS-R1. Y[Bpa2]DrmMS retained many of the DrmMS contact sites with DrmMS-R1, thus, mimicking the parent peptide consistent with the SAR data that established it is active in heart and gut. Y and Bpa2 are dark green and light green, respectively.
(TIF)

S3 Fig Y[Bpa4]DrmMS docked to DrmMS-R1. Y[Bpa4]DrmMS did not retain many of the DrmMS contact sites with DrmMS-R1, thus, it did not mimic the parent peptide but was consistent with the SAR data that established it as inactive in heart and gut. Bpa4 is dark red.
(TIF)

S4 Fig [5–10]DrmMS docked to DrmMS-R2. [5–10]DrmMS retained many of the DrmMS contact sites with DrmMS-R2, thus, it mimicked the parent peptide consistent with the SAR data that established it was active in heart. It likely acts through DrmMS-R2.
(TIF)

S5 Fig Y[Bpa2]DrmMS docked to DrmMS-R2. Y[Bpa2]DrmMS retained many of the DrmMS contact sites with DrmMS-R1, thus, it mimicked the parent peptide consistent with the SAR data that established it was active in heart and gut.
(TIF)

S6 Fig Y[Bpa4]DrmMS docked to DrmMS-R2. Y[Bpa4]DrmMS retained DrmMS interactions, however, many of the interactions were made by different ligand residues and did not retain the same physiochemical character, consistent with SAR data that established it had diminished activity in heart and gut.
(TIF)

S7 Fig [7–10]RhpMS docked to RhpMS-R. [7–10]RhpMS interacted with RhpMS contacts near TM5 but failed to fill the pocket or retain additional RhpMS contacts, suggesting that [7–10]RhpMS would not act through RhpMS-R.
(TIF)

S8 Fig DrmMS docked to RhpMS-R. DrmMS docked to RhpMS-R with F7, L8, and F10 forming extensive hydrophobic interactions near TM5. D4, H5, and R9 generated an ionic network that spanned from TM3 to TM7.
(TIF)

S9 Fig [4–10]RhpMS, [5–10]RhpMS, and [6–10]RhpMS dose-response effects on *R*. *prolixus* heart. The EC50 values were 32 nM, 31 nM, and 31 nM, respectively. The y-axis is the effect of [4–10]RhpMS (top), [5–10]RhpMS (middle), and [6–10]RhpMS (bottom) on heart rate as percent of saline (100%). The x-axis is concentration as molarity, M. Mean values ± S.E.M are reported.
(TIF)

S10 Fig RhpMS and N-terminal truncated analogs on heart: competition curves. The EC_50_ values were 24 nM, 110 nM, and 54 nM, respectively. The y-axis is the effect of RhpMS + [4–10]RhpMS (top), [5–10]RhpMS (middle), and [6–10]RhpMS (bottom) on heart rate as percent of saline (100%). The x-axis is concentration as molarity, M. Mean values ± S.E.M are reported. N = 10 animals for each experiment.
(TIF)

S1 Table [7–10]DrmMS ligand-receptor contact sites on DrmMS-R1^a^. ^a^ Residues numbered 1–10 are in DrmMS or RhpMS. (NH) and (CO) indicate that the residue backbone group was contacted. In the case in which a residue was contacted twice by the backbone or side chain of the same ligand residue, O and H (backbone atoms), OH (hydroxyl of Y), and CO (carbonyl of Bpa) are used to distinguish the contacts.
(DOCX)

S2 Table Y[Bpa2]DrmMS contact sites on DrmMS-R1^a^. ^a^ Residues numbered 1–10 are in DrmMS or RhpMS. (NH) and (CO) indicate that the residue backbone group was contacted. In the case in which a residue was contacted twice by the backbone or side chain of the same ligand residue, O and H (backbone atoms), OH (hydroxyl of Y), and CO (carbonyl of Bpa) are used to distinguish the contacts.
(DOCX)

S3 Table Y[Bpa4]DrmMS contact sites on DrmMS-R1^a^. ^a^ Residues numbered 1–10 are in DrmMS or RhpMS. (NH) and (CO) indicate that the residue backbone group was contacted. In the case in which a residue was contacted twice by the backbone or side chain of the same ligand residue, O and H (backbone atoms), OH (hydroxyl of Y), and CO (carbonyl of Bpa) are used to distinguish the contacts.
(DOCX)

S4 Table [5–10]DrmMS ligand-receptor contact sites on DrmMS-R2^a^. ^a^ Residues numbered 1–10 are in DrmMS or RhpMS. (NH) and (CO) indicate that the residue backbone group was contacted. In the case in which a residue was contacted twice by the backbone or side chain of the same ligand residue, O and H (backbone atoms), OH (hydroxyl of Y), and CO (carbonyl of Bpa) are used to distinguish the contacts.
(DOCX)

S5 Table Y[Bpa2]DrmMS contact sites on DrmMS-R2^a^. ^a^ Residues numbered 1–10 are in DrmMS or RhpMS. (NH) and (CO) indicate that the residue backbone group was contacted. In the case in which a residue was contacted twice by the backbone or side chain of the same ligand residue, O and H (backbone atoms), OH (hydroxyl of Y), and CO (carbonyl of Bpa) are used to distinguish the contacts.
(DOCX)

S6 Table Y[Bpa4]DrmMS contact sites on DrmMS-R2^a^. ^a^ Residues numbered 1–10 are in DrmMS or RhpMS. (NH) and (CO) indicate that the residue backbone group was contacted. In the case in which a residue was contacted twice by the backbone or side chain of the same ligand residue, O and H (backbone atoms), OH (hydroxyl of Y), and CO (carbonyl of Bpa) are used to distinguish the contacts.
(DOCX)

S7 Table [7–10]RhpMS ligand-receptor contact sites on RhpMS-R^a^. ^a^ Residues numbered 1–10 are in DrmMS or RhpMS. (NH) and (CO) indicate that the residue backbone group was contacted. In the case in which a residue was contacted twice by the backbone or side chain of the same ligand residue, O and H (backbone atoms), OH (hydroxyl of Y), and CO (carbonyl of Bpa) are used to distinguish the contacts.
(DOCX)

S8 Table DrmMS ligand-receptor contact sites on RhpMS-R^a^. ^a^ Residues numbered 1–10 are in DrmMS or RhpMS. (NH) and (CO) indicate that the residue backbone group was contacted. In the case in which a residue was contacted twice by the backbone or side chain of the same ligand residue, O and H (backbone atoms), OH (hydroxyl of Y), and CO (carbonyl of Bpa) are used to distinguish the contacts.
(DOCX)
