# Supplementary figures and images for: Cardiac Contractility Structure-Activity Relationship and Ligand-Receptor Interactions; the Discovery Of Unique and Novel Molecular Switches in Myosuppressin Signaling
Source: PLoS One. 2015 Mar 20;10(3):e0120492. doi: 10.1371/journal.pone.0120492 (PMC4368603; doi:10.1371/journal.pone.0120492)

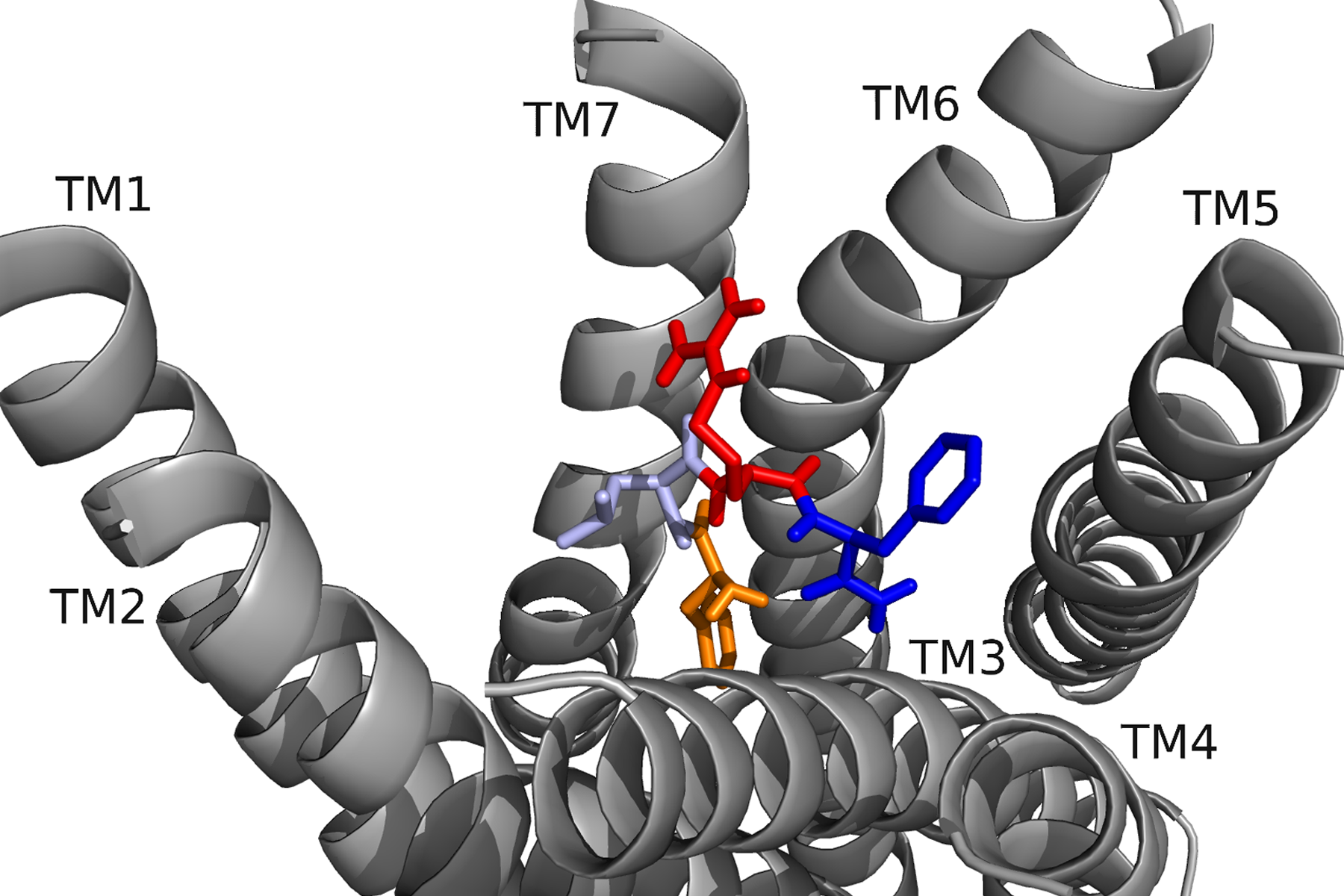

Supplement: S1 Fig — [7–10]DrmMS retained few contacts of DrmMS and the hydrophobic network between F7 and L8 was weakened in comparison to the parent peptide, consistent with the inactivity of the analog. (TIF) [file pone.0120492.s001.tif]

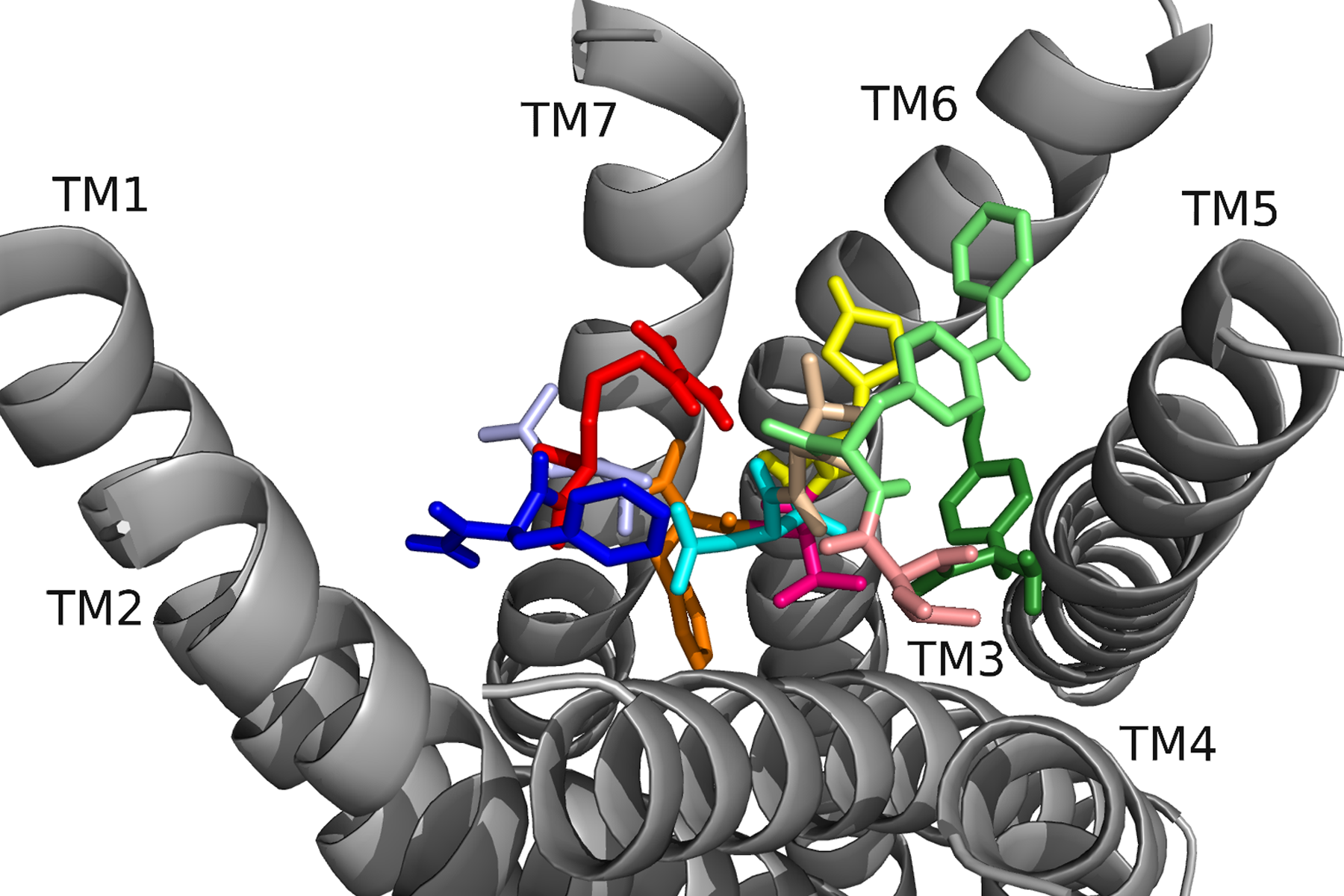

Supplement: S2 Fig — Y[Bpa2]DrmMS retained many of the DrmMS contact sites with DrmMS-R1, thus, mimicking the parent peptide consistent with the SAR data that established it is active in heart and gut. Y and Bpa2 are dark green and light green, respectively. (TIF) [file pone.0120492.s002.tif]

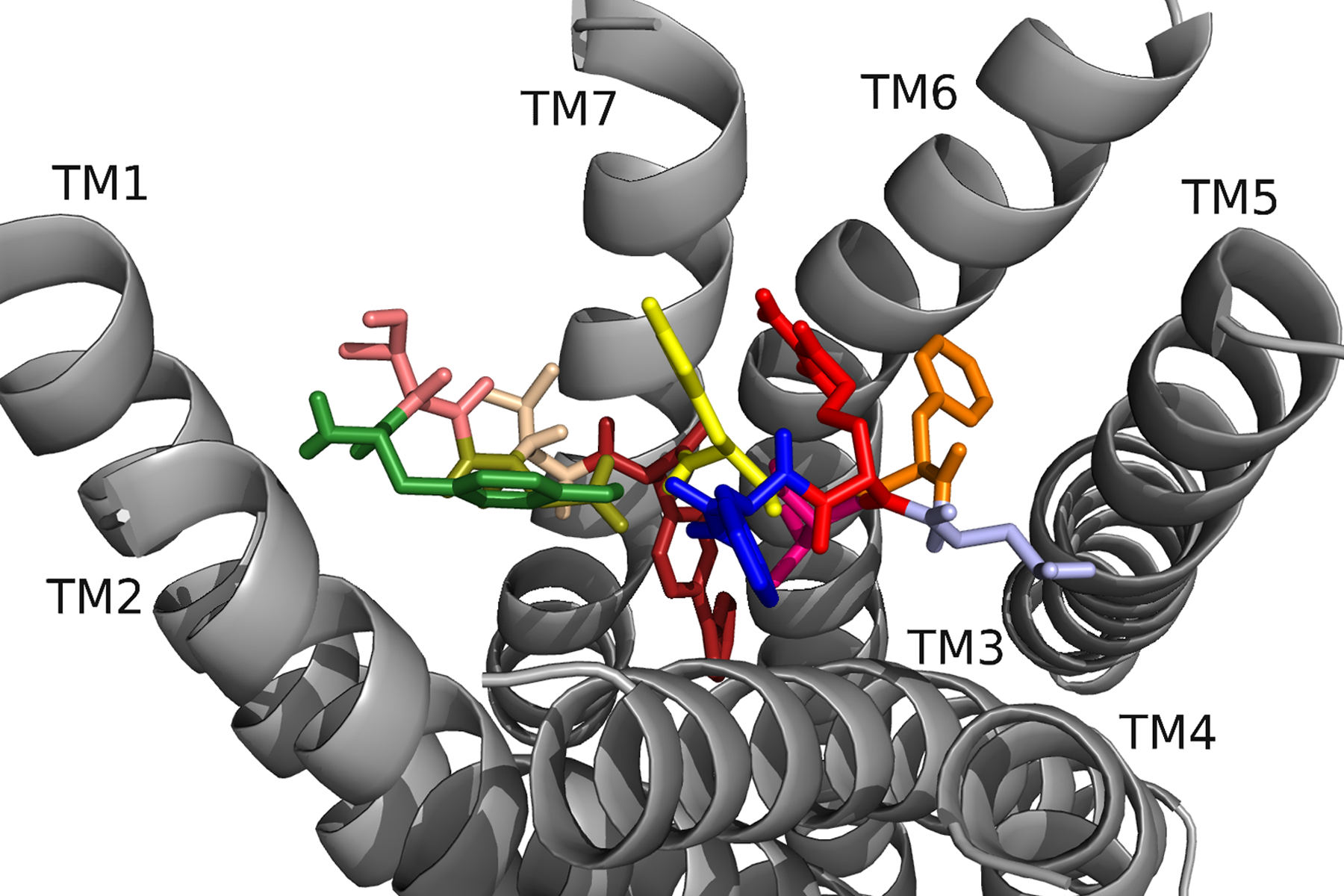

Supplement: S3 Fig — Y[Bpa4]DrmMS did not retain many of the DrmMS contact sites with DrmMS-R1, thus, it did not mimic the parent peptide but was consistent with the SAR data that established it as inactive in heart and gut. Bpa4 is dark red. (TIF) [file pone.0120492.s003.tif]

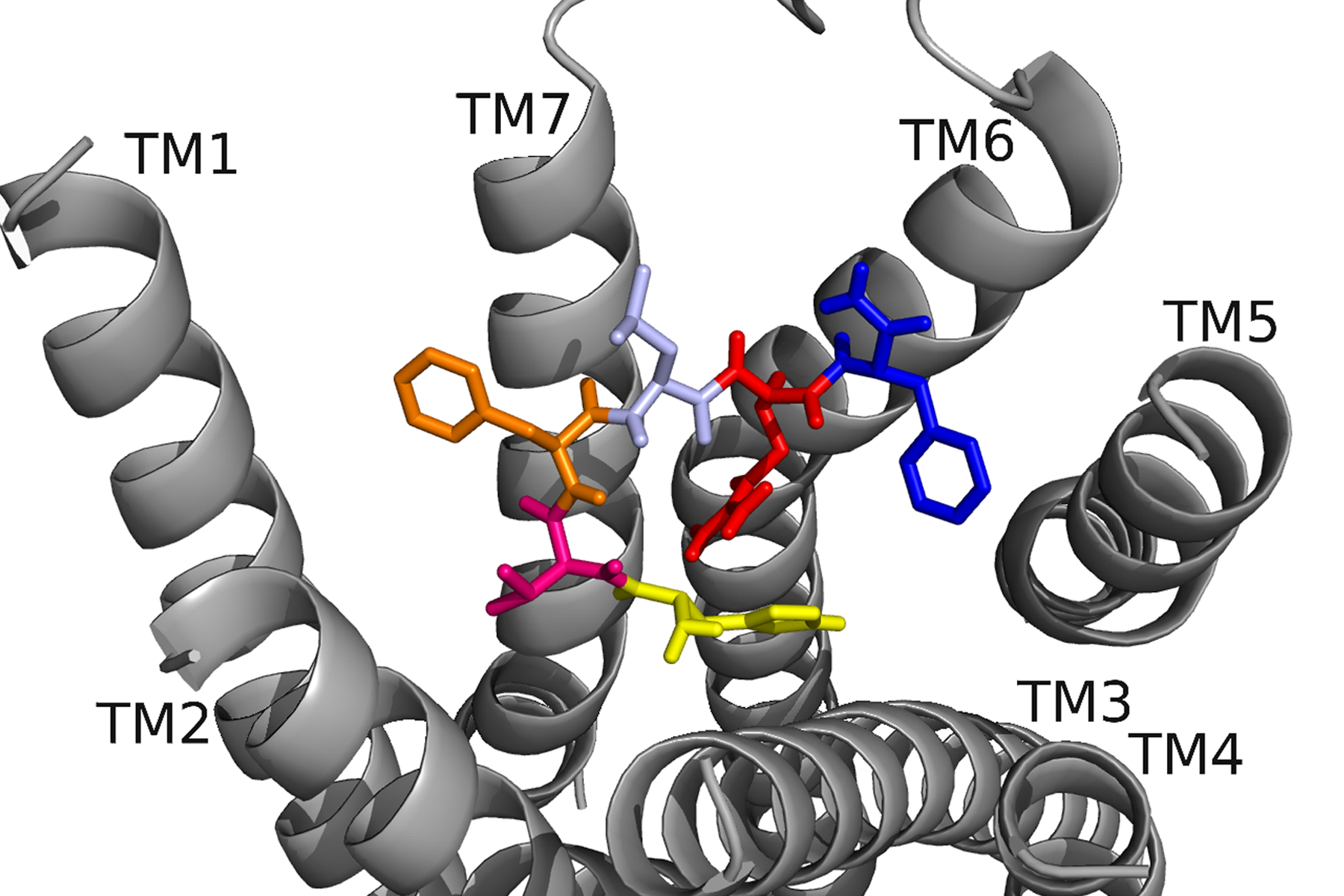

Supplement: S4 Fig — [5–10]DrmMS retained many of the DrmMS contact sites with DrmMS-R2, thus, it mimicked the parent peptide consistent with the SAR data that established it was active in heart. It likely acts through DrmMS-R2. (TIF) [file pone.0120492.s004.tif]

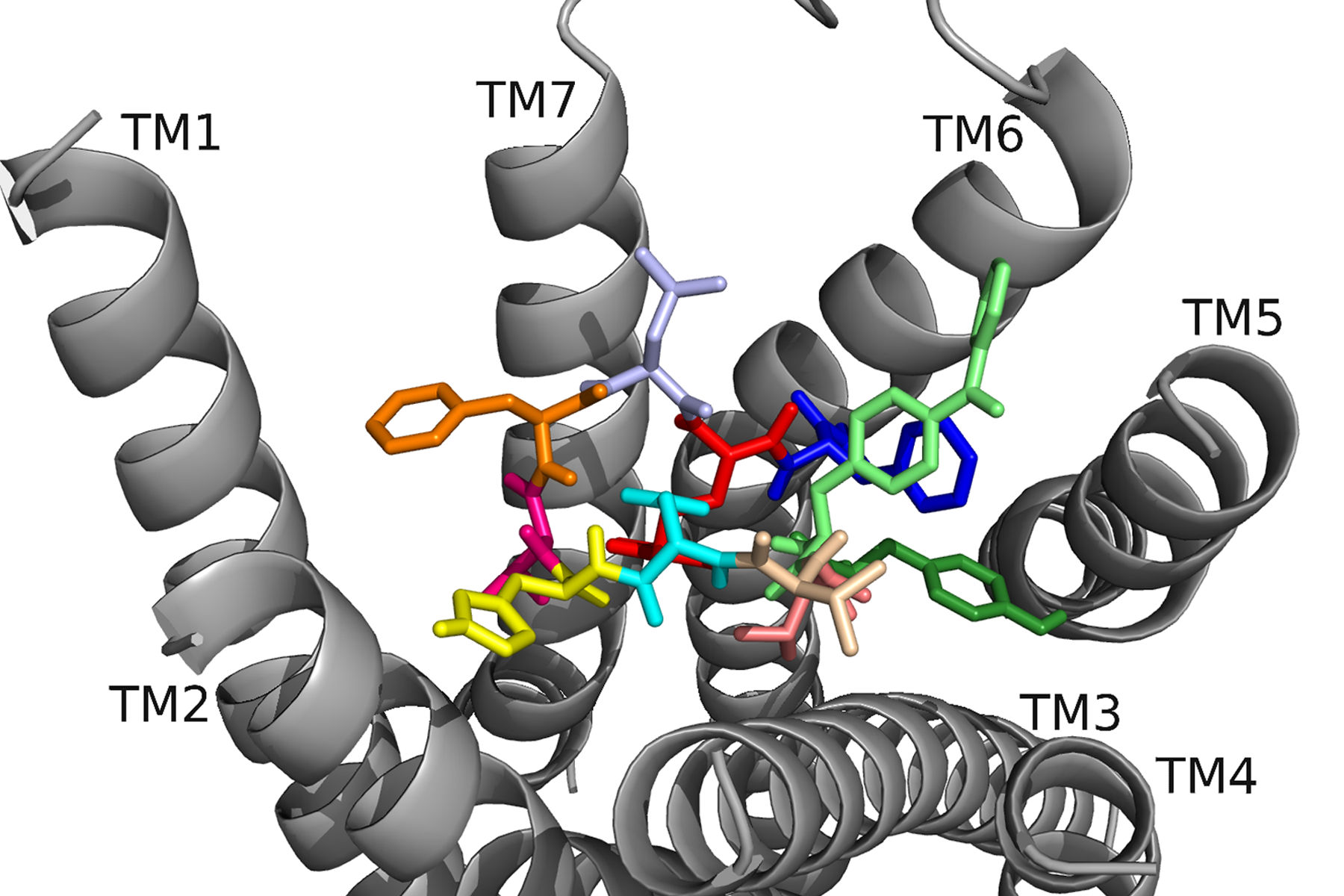

Supplement: S5 Fig — Y[Bpa2]DrmMS retained many of the DrmMS contact sites with DrmMS-R1, thus, it mimicked the parent peptide consistent with the SAR data that established it was active in heart and gut. (TIF) [file pone.0120492.s005.tif]

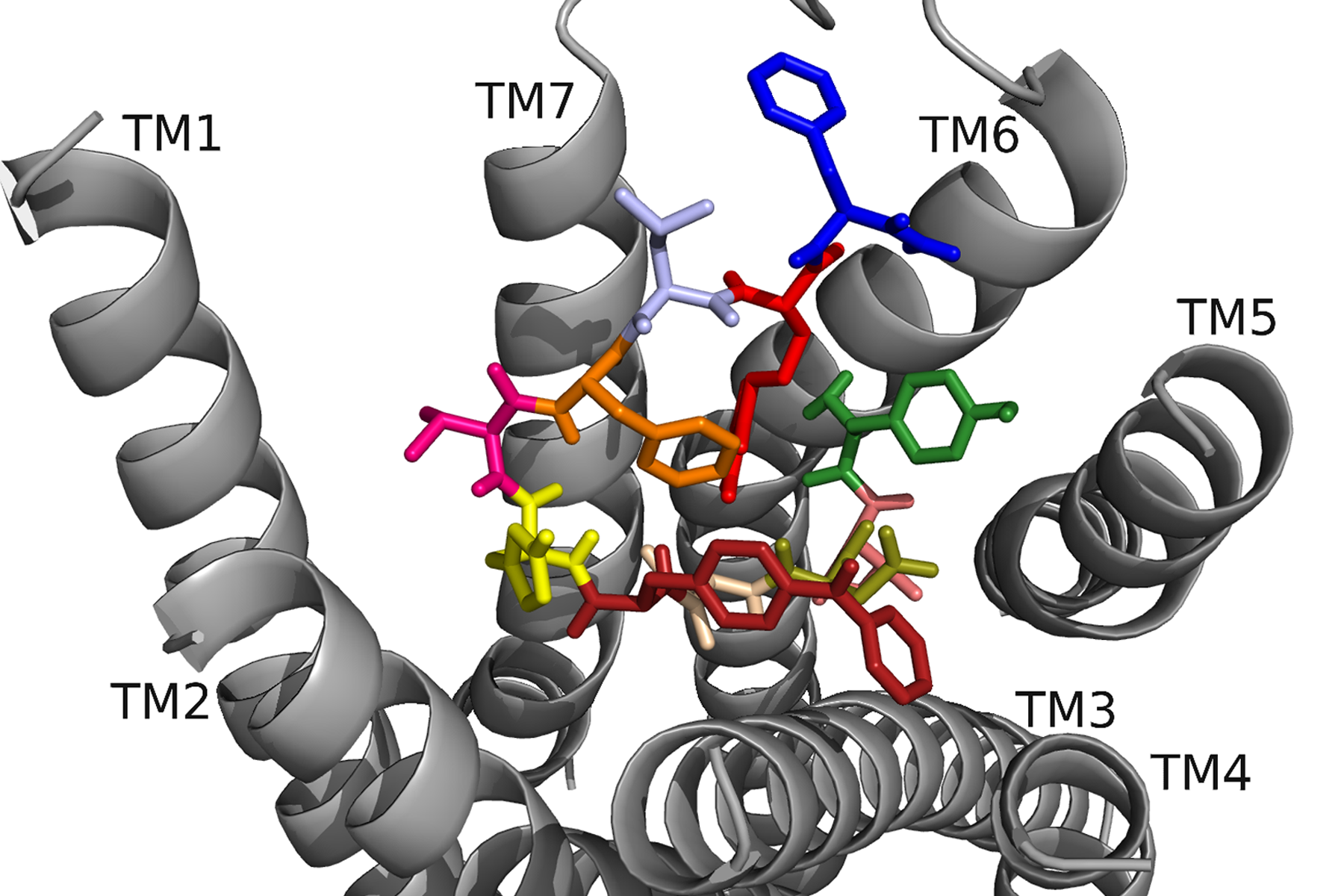

Supplement: S6 Fig — Y[Bpa4]DrmMS retained DrmMS interactions, however, many of the interactions were made by different ligand residues and did not retain the same physiochemical character, consistent with SAR data that established it had diminished activity in heart and gut. (TIF) [file pone.0120492.s006.tif]

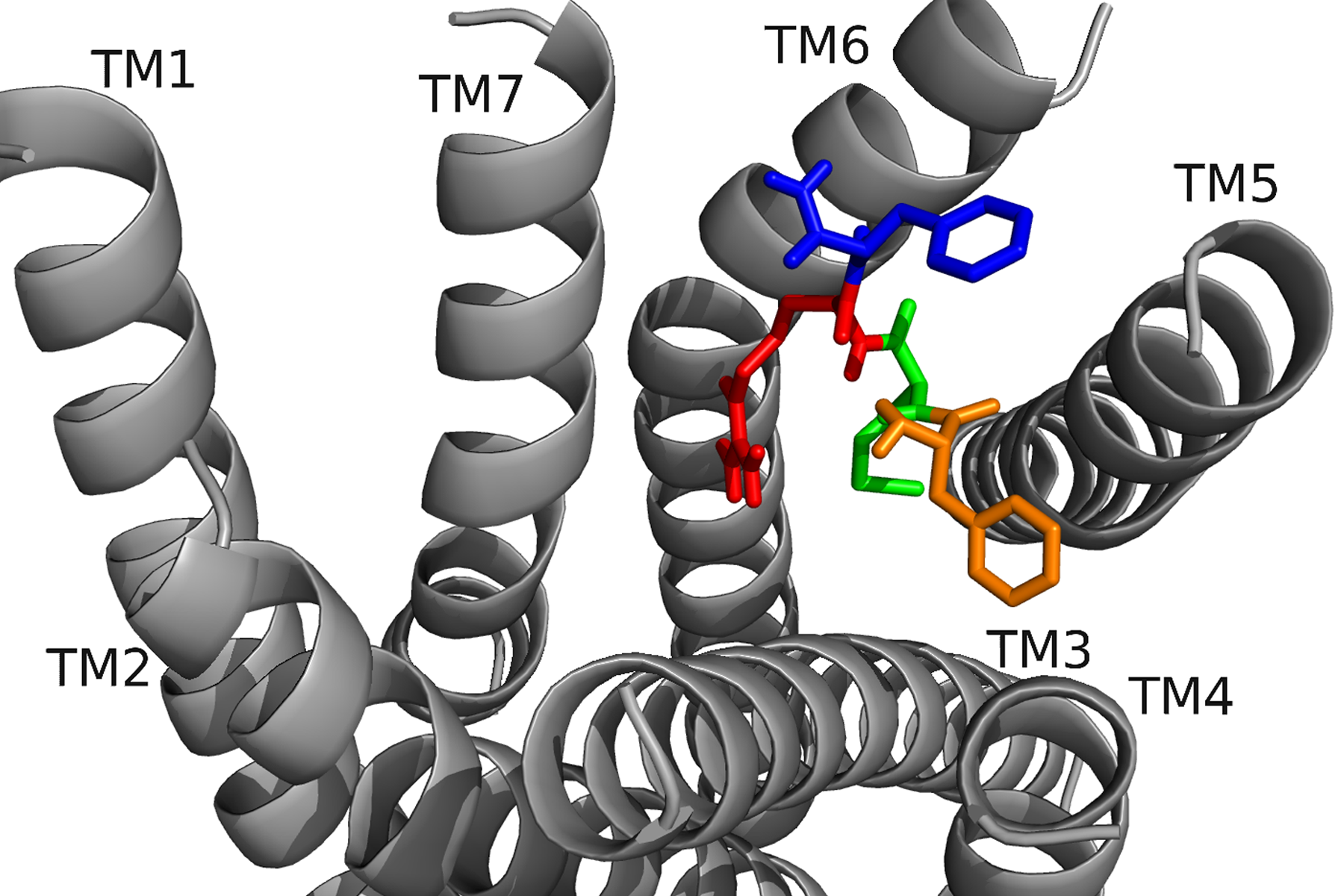

Supplement: S7 Fig — [7–10]RhpMS interacted with RhpMS contacts near TM5 but failed to fill the pocket or retain additional RhpMS contacts, suggesting that [7–10]RhpMS would not act through RhpMS-R. (TIF) [file pone.0120492.s007.tif]

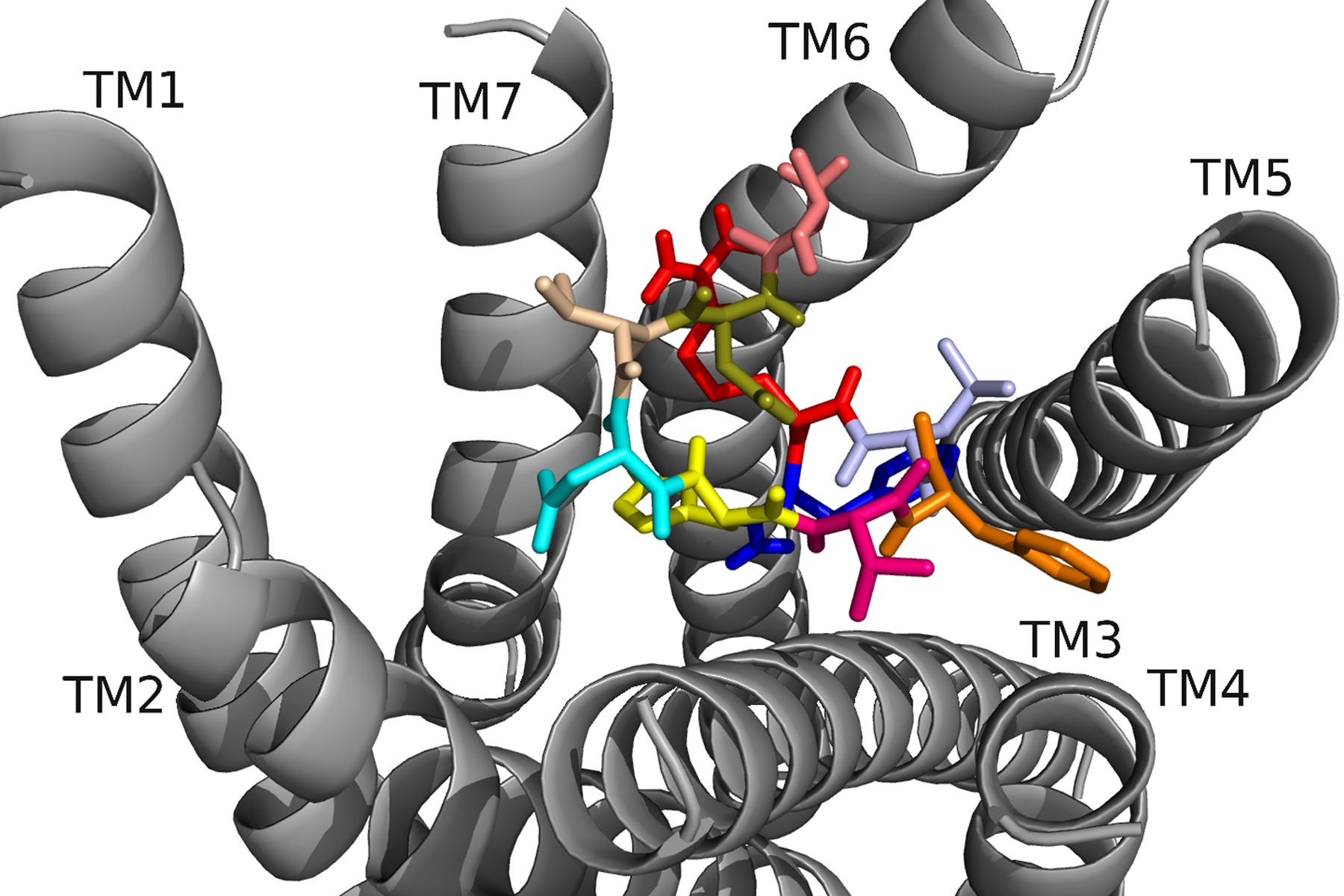

Supplement: S8 Fig — DrmMS docked to RhpMS-R with F7, L8, and F10 forming extensive hydrophobic interactions near TM5. D4, H5, and R9 generated an ionic network that spanned from TM3 to TM7. (TIF) [file pone.0120492.s008.tif]

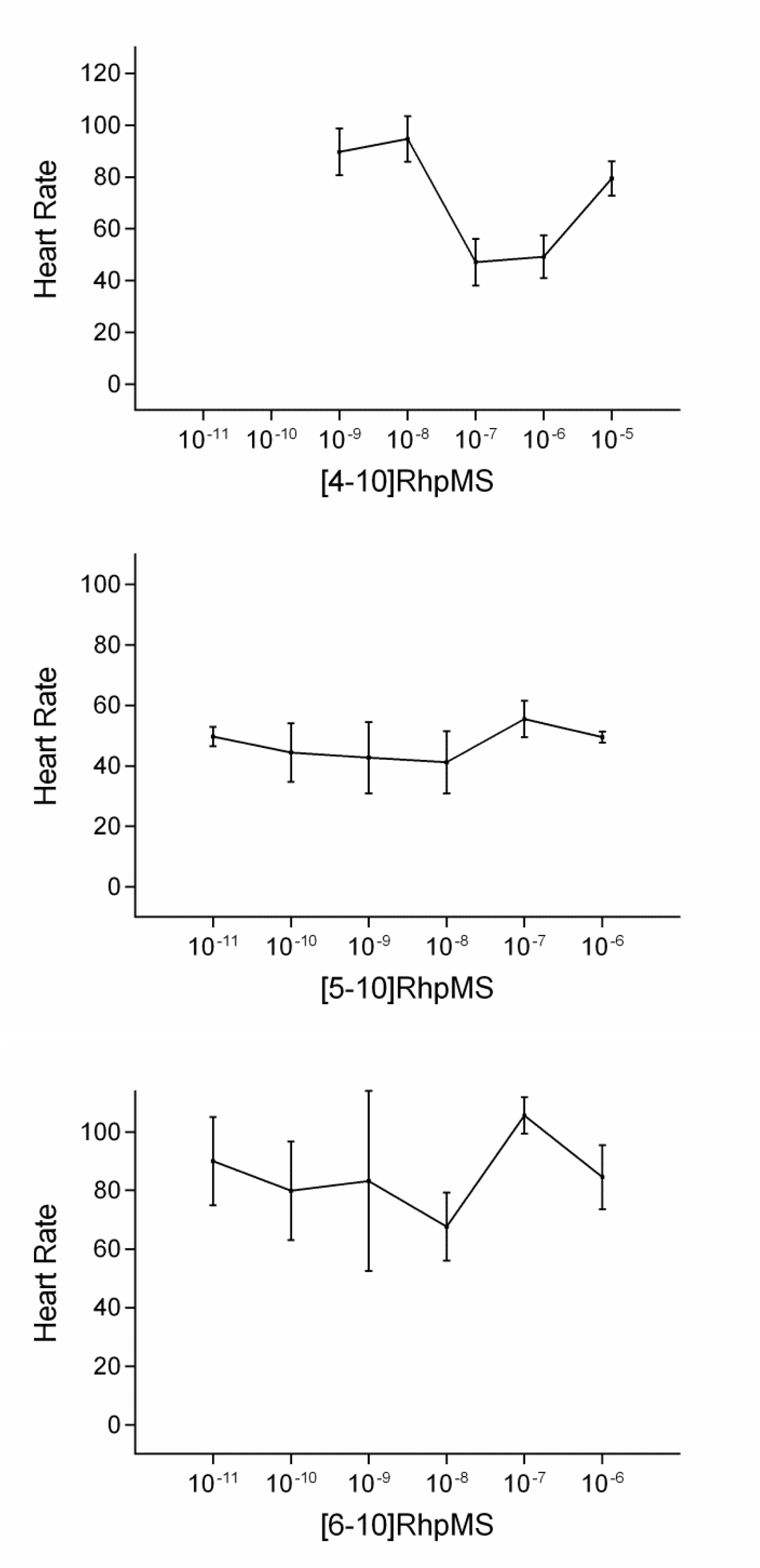

Supplement: S9 Fig — The EC50 values were 32 nM, 31 nM, and 31 nM, respectively. The y-axis is the effect of [4–10]RhpMS (top), [5–10]RhpMS (middle), and [6–10]RhpMS (bottom) on heart rate as percent of saline (100%). The x-axis is concentration as molarity, M. Mean values ± S.E.M are reported. (TIF) [file pone.0120492.s009.tif]

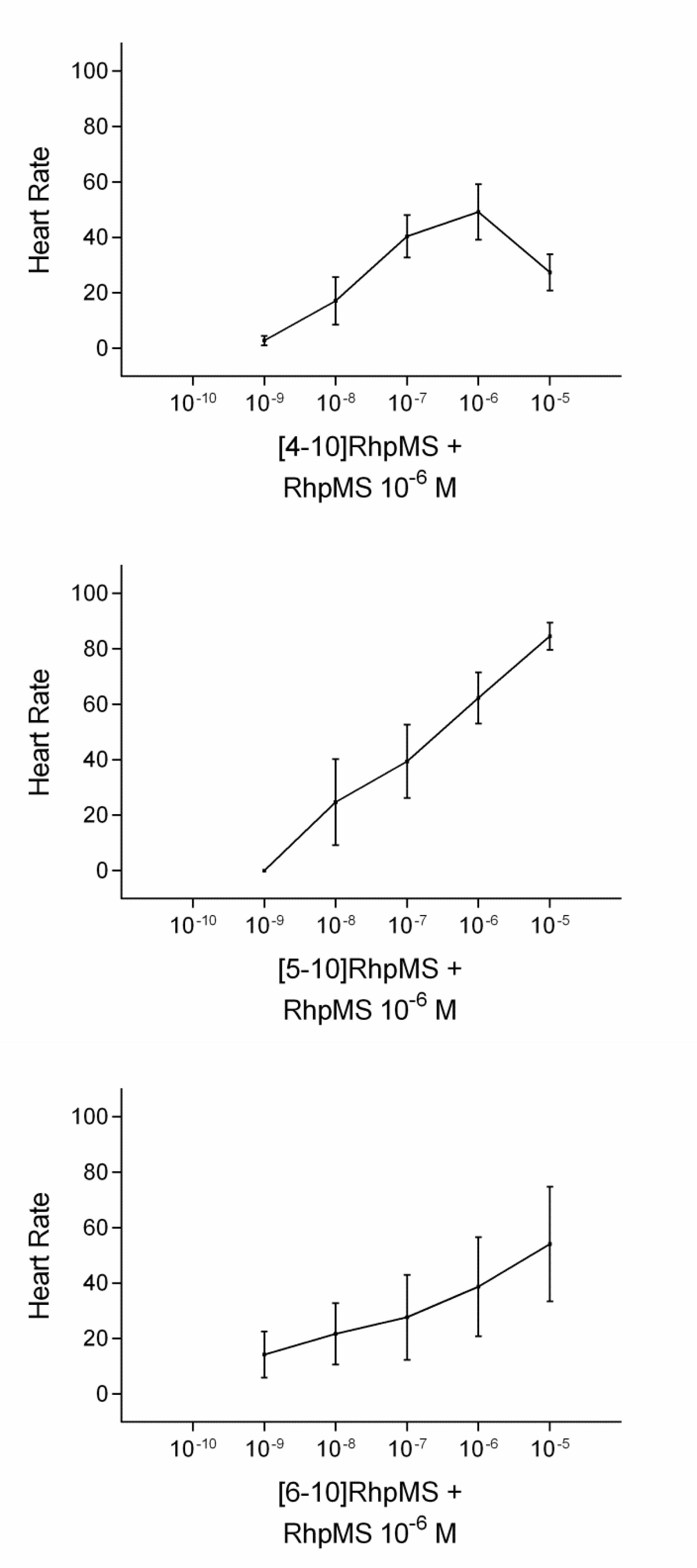

Supplement: S10 Fig — The EC50 values were 24 nM, 110 nM, and 54 nM, respectively. The y-axis is the effect of RhpMS + [4–10]RhpMS (top), [5–10]RhpMS (middle), and [6–10]RhpMS (bottom) on heart rate as percent of saline (100%). The x-axis is concentration as molarity, M. Mean values ± S.E.M are reported. N = 10 animals for each experiment. (TIF) [file pone.0120492.s010.tif]
