# Supplementary material for: Cardiac Contractility Structure-Activity Relationship and Ligand-Receptor Interactions; the Discovery Of Unique and Novel Molecular Switches in Myosuppressin Signaling
Source: PLoS One. 2015 Mar 20;10(3):e0120492. doi: 10.1371/journal.pone.0120492 (PMC4368603; doi:10.1371/journal.pone.0120492)
Supplement: S1 Table — a Residues numbered 1–10 are in DrmMS or RhpMS. (NH) and (CO) indicate that the residue backbone group was contacted. In the case in which a residue was contacted twice by the backbone or side chain of the same ligand residue, O and H (backbone atoms), OH (hydroxyl of Y), and CO (carbonyl of Bpa) are used to distinguish the contacts. (DOCX) [file pone.0120492.s011.docx]

**S1 Table. [7-10]DrmMS ligand-receptor contact sites on DrmMS-R1^a^.**

| F | Side chain | V73 | 3.8 Å |
| --- | --- | --- | --- |
|  |  | L115 | 4.9 Å |
|  |  | H116 | 4.0 Å |
|  |  | F361 | 4.2 Å |
|  |  | L399 | 4.0 Å |
|  | Backbone | H116 | 4.0 Å |
|  |  | Q368 | 4.1 Å |
|  |  | L8 | (NH) 2.2 Å |
|  |  | R9 | (NH) 2.7 Å |
|  |  | F10 | (CO) 2.1 Å |
| L | Side chain | Y77 | 3.4 Å |
|  |  | T81 | 4.7 Å |
|  |  | L399 | 3.6 Å |
|  | Backbone | Q368 | 3.2 Å |
|  |  | F7 | (NH) 2.2 Å |
|  |  | R9 | 2.5 Å |
| R | Side chain | D392 | 2.1 Å |
|  |  | D395 | 2.6 Å |
|  |  | L8 | (CO) 2.5 Å |
|  | Backbone | Q368 | 2.9 Å |
|  |  | F7 | (NH) 2.7 Å |
|  |  | F10 | (CO) 3.7 Å |
| F | Side chain | F273 | 3.6 Å |
|  |  | Y276 | 3.5 Å |
|  | Backbone | F7 | (NH) 2.1 Å |
|  |  | R9 | (NH) 3.7 Å |
| NH_2_ |  | Y276 | 2.1 Å |

^a^Residues numbered 1-10 are in DrmMS or RhpMS. (NH) and (CO) indicate that the residue backbone group was contacted. In the case in which a residue was contacted twice by the backbone or side chain of the same ligand residue, O and H (backbone atoms), OH (hydroxyl of Y), and CO (carbonyl of Bpa) are used to distinguish the contacts.
