# Supplementary material for: Cardiac Contractility Structure-Activity Relationship and Ligand-Receptor Interactions; the Discovery Of Unique and Novel Molecular Switches in Myosuppressin Signaling
Source: PLoS One. 2015 Mar 20;10(3):e0120492. doi: 10.1371/journal.pone.0120492 (PMC4368603; doi:10.1371/journal.pone.0120492)
Supplement: S2 Table — a Residues numbered 1–10 are in DrmMS or RhpMS. (NH) and (CO) indicate that the residue backbone group was contacted. In the case in which a residue was contacted twice by the backbone or side chain of the same ligand residue, O and H (backbone atoms), OH (hydroxyl of Y), and CO (carbonyl of Bpa) are used to distinguish the contacts. (DOCX) [file pone.0120492.s012.docx]

**S2 Table. Y[Bpa2]DrmMS contact sites on DrmMS-R1^a^.**

| Y | Side chain | T272 | 4.0 Å |
| --- | --- | --- | --- |
|  |  | F273 | 3.7 Å |
|  |  | Y276 | 3.4 Å |
|  |  | Bpa2 | 4.5 Å, OH 3.8 Å |
|  |  | V3 | 4.4 Å |
|  |  | H5 | 2.7 Å |
|  |  | V6 | 5.0 Å |
|  | Backbone | Q113 | 2.9 Å |
|  |  | V3 | (NH) 3.9 Å |
| T | side chain | S167 | 4.0 Å |
|  | Backbone | -- |  |
| Bpa | Side chain | Q269 | 3.1 Å |
|  |  | F273 | 3.6 Å |
|  |  | Y | 4.5 Å, CO 3.8 Å |
|  |  | V3 | 3.6 Å |
|  |  | H5 | 3.6 Å |
|  | Backbone | V3 | (CO) 3.8 Å |
| V | Side chain | Y | 4.4 Å |
|  |  | Bpa2 | 3.6 Å |
|  |  | H5 | 4.1 Å |
|  | Backbone | Q113 | 3.2 Å |
|  |  | Y | (CO) 3.9 Å |
|  |  | Bpa2 | (NH) 3.8 Å |
| D | Side chain | Y77 | 3.2 Å |
|  |  | H5 | (NH) 3.9 Å |
|  |  | R9 | 3.6 Å |
|  | Backbone | Q113 | 3.7 Å |
| H | Side chain | F273 | 3.6 Å |
|  |  | M371 | 4.0 Å |
|  |  | D395 | 3.6 Å |
|  |  | Y | 2.7 Å |
|  |  | Bpa2 | 3.6 Å |
|  |  | V3 | 4.1 Å |
|  | Backbone | K281 | 3.7 Å |
|  |  | Q368 | 3.8 Å |
|  |  | D4 | 3.9 Å |
| V | Side chain | A112 | 4.3 Å |
|  |  | H116 | 3.5 Å |
|  |  | T117 | 5.0 Å |
|  |  | Y276 | 4.7 Å |
|  |  | Y | 5.0 Å |
|  |  | F7 | 4.0 Å |
|  | Backbone | H116 | 3.4 Å |
|  |  | Q368 | 3.9 Å |
|  |  | F7 | (CO) 3.7 Å |
| F | Side chain | V73 | 3.5 Å |
|  |  | Y77 | 5.1 Å |
|  |  | A112 | 4.5 Å |
|  |  | L115 | 4.5 Å |
|  |  | H116 | 3.9 Å |
|  |  | L399 | 3.7 Å |
|  |  | V6 | 4.0 Å |
|  | Backbone | Q368 | 3.4 Å |
|  |  | V6 | (NH) 3.7 Å |
|  |  | L8 | (CO) 3.8 Å |
|  |  | R9 | 3.4 Å |
| L | Side chain | H28 | 4.6 Å |
|  |  | Y85 | 4.7 Å |
|  |  | I396 | 3.6 Å |
|  |  | L399 | 3.8 Å |
|  | Backbone | Y77 | 4.0 Å |
|  |  | F7 | (NH) 3.8 Å |
|  |  | R9 | 3.8 Å |
| R | Side chain | Q368 | 4.0 Å |
|  |  | D392 | 3.0 Å |
|  |  | D395 | 3.3 Å |
|  |  | D4 | 3.6 Å |
|  |  | F7 | (CO) 2.4 Å |
|  |  | L8 | (CO) 3.8 Å |
|  | Backbone | Y77 | 2.8 Å |
|  |  | H108 | 3.1 Å |
|  |  | NH_2_ | 2.6 Å |
| F | Side chain | W101 | 4.6 Å |
|  |  | I105 | 3.7 Å |
|  |  | H108 | 4.0 Å |
|  | Backbone | -- |  |
| NH_2_ |  | H108 | 3.3 Å |
|  |  | R9 | (CO) 2.6 Å |

^a^Residues numbered 1-10 are in DrmMS or RhpMS. (NH) and (CO) indicate that the residue backbone group was contacted. In the case in which a residue was contacted twice by the backbone or side chain of the same ligand residue, O and H (backbone atoms), OH (hydroxyl of Y), and CO (carbonyl of Bpa) are used to distinguish the contacts.
