# Supplementary material for: Cardiac Contractility Structure-Activity Relationship and Ligand-Receptor Interactions; the Discovery Of Unique and Novel Molecular Switches in Myosuppressin Signaling
Source: PLoS One. 2015 Mar 20;10(3):e0120492. doi: 10.1371/journal.pone.0120492 (PMC4368603; doi:10.1371/journal.pone.0120492)
Supplement: S6 Table — a Residues numbered 1–10 are in DrmMS or RhpMS. (NH) and (CO) indicate that the residue backbone group was contacted. In the case in which a residue was contacted twice by the backbone or side chain of the same ligand residue, O and H (backbone atoms), OH (hydroxyl of Y), and CO (carbonyl of Bpa) are used to distinguish the contacts. (DOCX) [file pone.0120492.s016.docx]

**S6 Table. Y[Bpa4]DrmMS contact sites on DrmMS-R2^a^.**

| Y | Side chain | W165 | 4.7 Å |
| --- | --- | --- | --- |
|  |  | R277 | 2.7 Å |
|  |  | N278 | 3.3 Å |
|  |  | F281 | 3.4 Å |
|  |  | G376 | 4.3 Å |
|  |  | V380 | 4.1 Å |
|  | Backbone | R9 | 2.9 Å |
| T | Side chain | H114 | 3.9 Å |
|  |  | F281 | 4.4 Å |
|  |  | K289 | 3.4 Å |
|  | Backbone | V3 | (NH) 2.9 Å |
| D | Side chain | Q111 | 3.0 Å |
|  |  | Bpa4 | (NH) 3.9 Å |
|  | Backbone | R9 | 3.4 Å |
| V | Side chain | Y75 | 4.3 Å |
|  |  | H106 | 4.3 Å |
|  |  | P110 | 3.9 Å |
|  |  | H114 | 4.5 Å |
|  |  | F7 | 4.7 Å |
|  | Backbone | H106 | 2.8 Å |
|  |  | T1 | (CO) 2.9 Å |
| Bpa | Side chain | I103 | 3.7 Å |
|  |  | H106 | 5.2 Å |
|  |  | W165 | 4.0 Å |
|  |  | H5 | 4.0 Å |
|  |  | F7 | 3.7 Å |
|  | Backbone | H103 | 3.3 Å |
|  |  | D2 | (CO) 3.9 Å |
|  |  | H5 | (CO) 3.9 Å |
| H | Side chain | Y78 | 4.0 Å |
|  |  | D82 | 3.4 Å |
|  |  | Y83 | 5.0 Å |
|  |  | I103 | 3.9 Å |
|  |  | Bpa4 | 4.0 Å |
|  |  | V6 | 4.1 Å |
|  |  | F7 | 4.8 Å |
|  | Backbone | Y83 | 4.7 Å |
|  |  | Bpa4 | (NH) 3.9 Å |
| V | Side chain | Y22 | 4.7 Å |
|  |  | K23 | 4.7 Å |
|  |  | H26 | 4.3 Å |
|  |  | Y83 | 3.7 Å |
|  |  | H5 | 4.1 Å |
|  | Backbone | -- |  |
| F | Side chain | V3 | 4.7 Å |
|  |  | Bpa4 | 3.7 Å |
|  |  | H5 | 4.8 Å |
|  | Backbone | S398 | 3.7 Å |
| L | Side chain | Y391 | 4.1 Å |
|  |  | L392 | 3.7 Å |
|  |  | F10 | 3.6 Å |
|  | Backbone | F10 | (NH) 2.7 Å |
| R | Side chain | Q372 | 3.0 Å |
|  |  | D399 | 3.0 Å |
|  |  | Y | (CO) 2.9 Å |
|  |  | D2 | (CO) 3.4 Å |
|  | Backbone | N379 | 3.5 Å |
|  |  | Y391 | O 2.7 Å, H 3.5 Å |
|  |  | NH_2_ | 2.4 Å |
| F | Side chain | F386 | 3.6 Å |
|  |  | Y391 | 3.7 Å |
|  |  | L8 | 3.6 Å |
|  | Backbone | L8 | (CO) 2.7 Å |
| NH_2_ |  | R9 | (CO) 2.4 Å |

^a^Residues numbered 1-10 are in DrmMS or RhpMS. (NH) and (CO) indicate that the residue backbone group was contacted. In the case in which a residue was contacted twice by the backbone or side chain of the same ligand residue, O and H (backbone atoms), OH (hydroxyl of Y), and CO (carbonyl of Bpa) are used to distinguish the contacts.
