# Supplementary material for: Cardiac Contractility Structure-Activity Relationship and Ligand-Receptor Interactions; the Discovery Of Unique and Novel Molecular Switches in Myosuppressin Signaling
Source: PLoS One. 2015 Mar 20;10(3):e0120492. doi: 10.1371/journal.pone.0120492 (PMC4368603; doi:10.1371/journal.pone.0120492)
Supplement: S7 Table — a Residues numbered 1–10 are in DrmMS or RhpMS. (NH) and (CO) indicate that the residue backbone group was contacted. In the case in which a residue was contacted twice by the backbone or side chain of the same ligand residue, O and H (backbone atoms), OH (hydroxyl of Y), and CO (carbonyl of Bpa) are used to distinguish the contacts. (DOCX) [file pone.0120492.s017.docx]

**S7 Table. [7-10]RhpMS ligand-receptor contact sites on RhpMS-R^a^.**

| F | Side chain | L161 | 4.6 Å |
| --- | --- | --- | --- |
|  |  | P164 | 5.1 Å |
|  |  | Y213 | 3.7 Å |
|  | Backbone | R9 | (CO) 2.3 Å |
| M | Side chain | Y213 | 4.4 Å |
|  |  | I217 | 4.0 Å |
|  |  | G285 | 5.1 Å |
|  | Backbone | -- |  |
| R | Side chain | Q111 | 3.8 Å |
|  |  | H114 | 2.6 Å |
|  |  | E281 | 3.2 Å |
|  | Backbone | F7 | (NH) 2.3 Å |
| F | Side chain | V206 | 4.2 Å |
|  |  | F210 | 3.7 Å |
|  |  | I292 | 3.8 Å |
|  | Backbone | T291 | 3.4 Å |
| NH_2_ |  | T291 | 2.9 Å |

^a^Residues numbered 1-10 are in DrmMS or RhpMS. (NH) and (CO) indicate that the residue backbone group was contacted. In the case in which a residue was contacted twice by the backbone or side chain of the same ligand residue, O and H (backbone atoms), OH (hydroxyl of Y), and CO (carbonyl of Bpa) are used to distinguish the contacts.
