# Supplementary material for: Cardiac Contractility Structure-Activity Relationship and Ligand-Receptor Interactions; the Discovery Of Unique and Novel Molecular Switches in Myosuppressin Signaling
Source: PLoS One. 2015 Mar 20;10(3):e0120492. doi: 10.1371/journal.pone.0120492 (PMC4368603; doi:10.1371/journal.pone.0120492)
Supplement: S8 Table — a Residues numbered 1–10 are in DrmMS or RhpMS. (NH) and (CO) indicate that the residue backbone group was contacted. In the case in which a residue was contacted twice by the backbone or side chain of the same ligand residue, O and H (backbone atoms), OH (hydroxyl of Y), and CO (carbonyl of Bpa) are used to distinguish the contacts. (DOCX) [file pone.0120492.s018.docx]

**S8 Table. DrmMS ligand-receptor contact sites on RhpMS-R^a^.**

| T | Side chain | T291 | 3.9 Å |
| --- | --- | --- | --- |
|  |  | D2 | 4.1 Å, (CO) 3.1 Å |
|  | Backbone | T291 | 2.0 Å |
|  |  | R9 | O 2.7 Å, H 3.7 Å |
| D | Side chain | T1 | 4.1 Å |
|  |  | H5 | (NH) 2.4 Å |
|  | Backbone | T1 | 3.1 Å |
| V | Side chain | G307 | 4.7 Å |
|  | Backbone | D311 | 3.2 Å |
|  |  | R9 | 4.0 Å |
| D | Side chain | Y77 | 2.9 Å |
|  |  | H106 | 3.2 Å |
|  |  | H5 | 3.5 Å |
|  | Backbone | D311 | 3.9 Å |
|  |  | H5 | 4.0 Å |
| H | Side chain | Y77 | 3.7 Å |
|  |  | T110 | 3.6 Å |
|  |  | H114 | 3.7 Å |
|  |  | D311 | 2.3 Å |
|  |  | D4 | 3.5 Å, (NH) 4.0 Å |
|  | Backbone | D2 | 2.4 Å |
| V | Side chain | S107 | 3.8 Å |
|  |  | L165 | 3.1 Å |
|  |  | F7 | 3.6 Å |
|  | Backbone | Q111 | 3.4 Å |
| F | Side chain | P164 | 4.0 Å |
|  |  | L165 | 4.6 Å |
|  |  | Y213 | 3.9 Å |
|  |  | V6 | 3.6 Å |
|  | Backbone | Q111 | 2.8 Å |
| L | Side chain | F210 | 3.9 Å |
|  |  | Y213 | 3.6 Å |
|  |  | G285 | 5.1 Å |
|  |  | G288 | 4.9 Å |
|  |  | M289 | 4.7 Å |
|  |  | F10 | 3.3 Å |
|  | Backbone | D2 | 3.6 Å |
| R | Side chain | D311 | 3.7 Å |
|  |  | T1 | (CO) 2.7 Å, (NH) 3.7 Å |
|  |  | V3 | (NH) 4.0 Å |
|  | Backbone | -- |  |
| F | Side chain | H114 | 3.8 Å |
|  |  | I118 | 3.7 Å |
|  |  | Y213 | 3.9 Å |
|  |  | I217 | 3.6 Å |
|  |  | K218 | 3.6 Å |
|  |  | L8 | 3.3 Å |
|  | Backbone | H114 | H 2.4 Å, O 3.0 Å |
| NH_2_ |  | E281 | 3.2 Å |

^a^Residues numbered 1-10 are in DrmMS or RhpMS. (NH) and (CO) indicate that the residue backbone group was contacted. In the case in which a residue was contacted twice by the backbone or side chain of the same ligand residue, O and H (backbone atoms), OH (hydroxyl of Y), and CO (carbonyl of Bpa) are used to distinguish the contacts.
